# The role of the 3′-UTR of the chemokine receptor CCR2 and hnRNPA0 in regulating mRNA stability and subcellular distribution in human CD4^+^ T cells

**DOI:** 10.3389/fimmu.2025.1655273

**Published:** 2025-08-20

**Authors:** Yunus Yukselten, Hanan Wishah, Lingyun Wang, Richard E. Sutton

**Affiliations:** Department of Internal Medicine, Section of Infectious Diseases, Yale School of Medicine, New Haven, CT, United States

**Keywords:** CCR2 3’-UTR, hnRNPA0, post-transcriptional regulation, RNA-binding proteins, CRISPR-Cas9 KO, R5-tropic HIV co-receptor

## Abstract

**Introduction:**

CCR2, a chemokine receptor critical for immune cell migration, inflammation, and HIV infection, is regulated by poorly understood mechanisms.

**Methods:**

This study investigated the unusually long CCR2 3’-UTR’s role in post-transcriptional regulation.

**Results:**

The full-length 3′-UTR significantly inhibited reporter gene expression in primary CD4+ T cells and macrophages, likely mediated by RNA binding proteins (RBPs). HnRNPA0, was shown to bind directly to this region and influence CCR2 levels. When the RBP binding sites were mutagenized or the 3′- UTR removed using CRISPR-Cas9 and gRNAs, CCR2 mRNA and protein levels significantly increased. Cell fractionation experiments confirmed that these changes occurred in both the nucleus and cytoplasm. To directly test mRNA stability, we used a-amanitin and found that removing the 3′-UTR nearly doubled the half-life of CCR2 mRNA. Finally, pseudotyping studies revealed CCR2 functions as an HIV co-receptor at ~10% efficiency compared to CCR5.

**Discussion:**

These results show that the CCR2 3′-UTR plays an important role in post-transcriptional regulation and may provide a novel approach to regulating CCR2 activity in inflammatory or infectious diseases.

## 1 Introduction

The C-C chemokine receptor 2 (CCR2) is a membrane-bound protein predominantly expressed on immune cell subsets such as lymphocytes, monocytes, macrophages, and CD4^+^ T lymphocytes (1). CCR2 plays a central role in mediating inflammatory responses triggered by stress or tissue damage, primarily by directing the migration of bone marrow–derived monocytes and macrophages to the affected site. These monocytes differ from resident monocytes in protein expression levels, among other aspects, with shorter-lived monocytes expressing higher levels of CCR2 for directing the cell toward the site of injury (2). Similar to other chemokine co-receptors, CCR2 has a notable lack of specificity in terms of ligands. CCR2 primarily binds to CCL2, which is its most potent and selective ligand, but it can also recognize chemokines such as CCL7, CCL8, and CCL13. Similarly, CCL2 is not exclusive to CCR2, as it can associate with other receptors including CCR1, CCR4, and CCR11 (1, 3, 4).

Cardiac macrophages are classified according to their CCR2 expression status. The CCR2^+^ population is defined by high levels of CCR2, a receptor abundantly expressed on monocytes and macrophages that are actively recruited to inflamed or injured cardiac tissue. In contrast, CCR2 negative macrophages do not express CCR2 and represent tissue-resident macrophages that are not directly involved in monocyte recruitment (5). These macrophages produce detrimental oxidative products, potentially contributing to heart failure via collateral damage and adverse remodeling of the left ventricle (6, 7). Careful and selective inhibition of CCR2+ monocytes and macrophages at early stages of cardiac stress decreased pathological hypertrophy, fibrosis, and systolic dysfunction, suggesting that modulating CCR2+ macrophages and monocytes may be critical in avoiding or preventing heart failure (6).

Because of the relationship between T cells and macrophages, CCR2+ macrophages have been of interest in cardiac transplants regarding donor rejection. Expression of CCR2- macrophages in cardiac tissue, which is tied to reduced inflammation, has been linked to decreased rates of cardiac transplant rejection. CCR2+ macrophages, on the other hand, display the opposite effect. Normal levels of CCR2+ macrophages present in cardiac donor tissue have been linked to increased rates of transplant rejection (8). Recent studies highlight that cardiac macrophage subsets can be functionally classified based on CCR2 expression, with CCR2^+^ macrophages primarily involved in inflammatory recruitment and CCR2^−^ subsets contributing to tissue repair and homeostasis (9).

The absence of MyD88, which is crucial for triggering CCR2^+^ macrophage activation in donor organs, results in decreased CCR2 expression on the cell surface and diminishes T cell responses against the foreign tissue (10). In liver tumors, CCR2 KO or antagonism has been shown to suppress hepatocellular carcinoma progression by reducing the recruitment of macrophages linked to tumors (1). CCR2, through its interaction with CCL2, has been associated with disease outcomes in multiple cancer types, such as hepatic, prostatic, mammary, and colorectal malignancies (1, 11, 12). CCR2 ligation significantly influences the apoptosis pathway by activating the PI3K/Akt pathway. This activation results in an upregulation of survivin and a downregulation of autophagosome formation. In tumor cells that express CCR2, both apoptosis and autophagic cell death are suppressed, enabling them to survive under otherwise lethal conditions, such as nutrient starvation.

CCR2 also plays a role in cancer metastasis, with CCR2 KO’d cells metastasizing significantly less frequently (11). CCR2 expression has also been linked to Alzheimer’s disease or AD (13, 14). As a result of chronic brain inflammation symptomatic of AD, monocytes enter the brain. These CCR2-expressing monocytes are then though to assist in generating blood-derived microglia, which cluster around Aβ plaques, reducing plaque accumulation and delaying the adverse cognitive effects symptomatic of AD (2).

CCR2 may also function as a co-receptor of R5-tropic human immunodeficiency virus type 1 or HIV (15). In HIV-seropositive patients, HIV-infected PBMCs express increased levels of CCR2 and CCL2; the virus may induce CCR2 protein expression (16, 17). The open reading frames of human CCR5 and CCR2 are 73% similarity at the amino acid level and bind many of the same ligands (18). Whereas CCR5 is widely expressed in T lymphocytes, macrophages, granulocytes, DCs, microglia, astrocytes, neurons, fibroblasts, and epithelial, endothelial, CCR2 is primarily expressed on the cell surface of monocytes, NK cells, and T lymphocytes (17, 19).

HIV replicates in CD4+ T cells, causing immunodeficiency. One of the first cytokines found in acute HIV infection, even before peak viremia, is CCL2, which is found in CD14+ monocytes (20). CCL2 binds to CCR2, which results in its swift upregulation in CD4+ T cells, which paradoxically provides HIV with a greater number of target host cells (20). The inflammation thus caused by HIV recruits additional susceptible CD4+ T cells to aggregate near virus-infected cells (20). Additionally, central memory CD4+ T cells can be infected and then act as a latent reservoir for the virus, making HIV cure a virtual impossibility (21). Although its impact is substantially less pronounced than that of CCR5, CCR2 may function as a co-receptor for some strains of HIV and plays alternative roles in progression of disease (22, 23).

In the V64I polymorphism of CCR2, valine at position 64 in the first transmembrane region is replaced by isoleucine (22). This CCR2 variant is linked to a slower progression of HIV to AIDS, though the precise mechanism is unknown. The leading hypothesis is that this variant of CCR2 dimerizes more frequently with CXCR4, the X4-tropic HIV co-receptor compared to the wild-type allele, which reduces CXCR4 cell surface levels, resulting in less susceptibility to HIV (15, 24).

CCR5 and CCR2 both play a critical role in the complex regulatory network of the immune system (17, 25). The expression of these two receptors has a decisive effect, particularly on inflammation and immune response processes; precisely how post-transcriptional regulatory mechanisms control these genes is largely unknown. The 3’-untranslated region (3`-UTR) is downstream of the coding sequence of the messenger RNA; it is not translated into protein. Instead, it may post-transcriptionally regulate gene expression, often through direct interaction with RNA binding proteins or micro RNAs, to enhance or antagonize gene expression (26). Pre-mRNA splicing and polyadenylation, which begin when the polyadenylation signal is detected, define the length of the 3’-UTR (27–29). About 50% of all human genes produce different mRNA variants with variable 3′-UTRs, resulting from alternative splicing and polyadenylation—while still encoding the same protein (30, 31).

Micro RNAs (miRNAs) are short forms of RNA that regulate transcription by binding to partially complementary sequences on the 3’-UTR, resulting in short, double-stranded segments of RNA. When the 3’-UTR of CCR5 is bound to small interfering or siRNAs, CCR5 levels decrease and R5-tropic HIV infection is reduced (32). Often, the 3’-UTR can manifest with multiple isoforms due to polyadenylation or alternative cleavage sites (33). The 3’-UTR of CCR2 has also been linked to metastatic suppression in some cancers (34).

RNA-binding proteins (RBPs) play important roles throughout the mRNA life cycle by interacting with unique sequences of the target RNA. These roles include pre-mRNA splicing, polyadenylation, mRNA stability, nuclear export, cytoplasmic transport, mRNA translation, and localization (35, 36). The hnRNP A/B family has emerged as an important player in cellular RNA metabolism. Their extensive involvement in RNA biogenesis and transport is crucial in maintaining cellular homeostasis and regulating gene expression under normal and stressful conditions (36, 37). hnRNPA0 belongs to the hnRNP A/B subfamily, which includes isoforms A1, A2/B1, and A3. hnRNPA0 is unique in its function in post-transcriptional regulation, especially in inflammatory circumstances, in which MAPKAP-K2 phosphorylates it on Ser84 (38). Here, we examined the role of the 3’-UTR of CCR2 in post-transcriptional gene regulation and investigated the involvement of RBPs in this process. We elucidated post-transcriptional regulation of CCR2 by analyzing the different regions of its 3’-UTR, and we investigated the interactions of its 3’-UTR with RBPs and the effects on mRNA transcription, cellular distribution, and translation. In addition, CRISPR-Cas9-based genetic modifications and *in vitro* binding experiments of the 3’-UTR RNA were performed to demonstrate that hnRNPA0 contributes to the post-transcriptional regulation of CCR2. We also show that CCR2 can function as a co-receptor for R5-tropic HIV, at roughly 10% of the level of CCR5. These findings may provide the basis for new therapeutic strategies to modulate the expression of CCR2.

## 2 Materials and methods

### 2.1 Isolation of primary human CD4+ T cells

Peripheral blood mononuclear cells (PBMCs) from anonymized human donors were acquired from the New York Blood Center (Queens, NY). Leukocyte-enriched fractions were from normal, non-hospitalized, HIV seronegative, healthy donors. Yale Institutional Board Committee considered this an IRB-exempt protocol since the PBMC samples had no other identifiable information.

PBMCs were isolated according to established protocols, employing SepMate™ tubes and Lymphoprep™ (STEMCELL Technologies). The EasySep™ Human CD4^+^ T Cell Isolation Kit was used to purify CD4^+^ T cells by negative selection. Analysis of CD4 protein expression on the surface of the purified T cells was performed by flow cytometry. To do so, cells were incubated on ice for 1 hr in staining buffer containing human anti-CD4-APC antibody (BioLegend, San Diego, CA). Samples were analyzed by flow cytometry using an LSRII cytometer (BD Biosciences), and data were processed with FlowJo v10.9 (Ashland, OR). The isolated CD4^+^ T cells consistently demonstrated a purity greater than 95% based upon flow cytometry.

### 2.2 Stimulation of primary CD4+ T cells

CD4^+^ T cells were activated over a 72-hour period using immobilized anti-CD3 antibody (BioLegend) in combination with anti-CD28 (BioLegend) and IL-2 (100 IU/ml; STEMCELL). Following activation, cells were cultured at 37 °C with 5% CO_2_ in RPMI 1640 medium supplemented with 10% fetal bovine serum and rIL-2.

### 2.3 Derivation, activation, and culture of macrophages

PBMCs were cultured at a concentration of 1× 10^6^ cells/mL in 12-well plates using RPMI 1640 medium enriched with 10% fetal bovine serum (FBS; GIBCO, USA), antibiotics (100 U/mL penicillin, 100 µg/mL streptomycin), 2.5 µg/mL amphotericin B, and 10 µg/mL ciprofloxacin.

Cells were maintained in a humidified incubator at 37° C supplemented with 5% CO_2_. Monocytes were isolated by adhering to cell culture plates for 24 hours, followed by washing to remove non-adherent cells. Adherent cells were cultivated for five days in culture medium supplemented with 50 ng/mL recombinant human macrophage-colony stimulating factor (M-CSF; Biolegend), 10 ng/ml IL-4 (Sino Biological), and 10 ng/ml IL-10 (Sino Biological). Monocytes were then matured to macrophages for 6–8 days, with microscopic evidence of plate adherence, clustering, and cytosolic enlargement, with limited dendrite formation.

### 2.4 Plasmid production and luciferase assay

The ~0.7 kb CMV promoter/enhancer was inserted just upstream of firefly luciferase (FFLUC) in pGL3-Basic Vector (Promega) and served as the positive control. Full-length, wild-type CCR2 3’-UTR was PCR-amplified from BAC RPII-237g13 (CHORI) and cloned into pCR-Blunt II-TOPO vector (Invitrogen) and sequence-confirmed. The resultant recombinant clone was digested with restriction enzymes Eco53KI and EcoRV to obtain the full-length 3`-UTR insert. After gel extraction, the ~2.4 kbp DNA fragment was then blunt-ligated in the unique XbaI site of pGL3-CMV- FFLUC, immediately downstream of the stop codon of FFLUC and upstream of the poly(A) addition site. This clone was named FlUTR.

**Figure 1:**
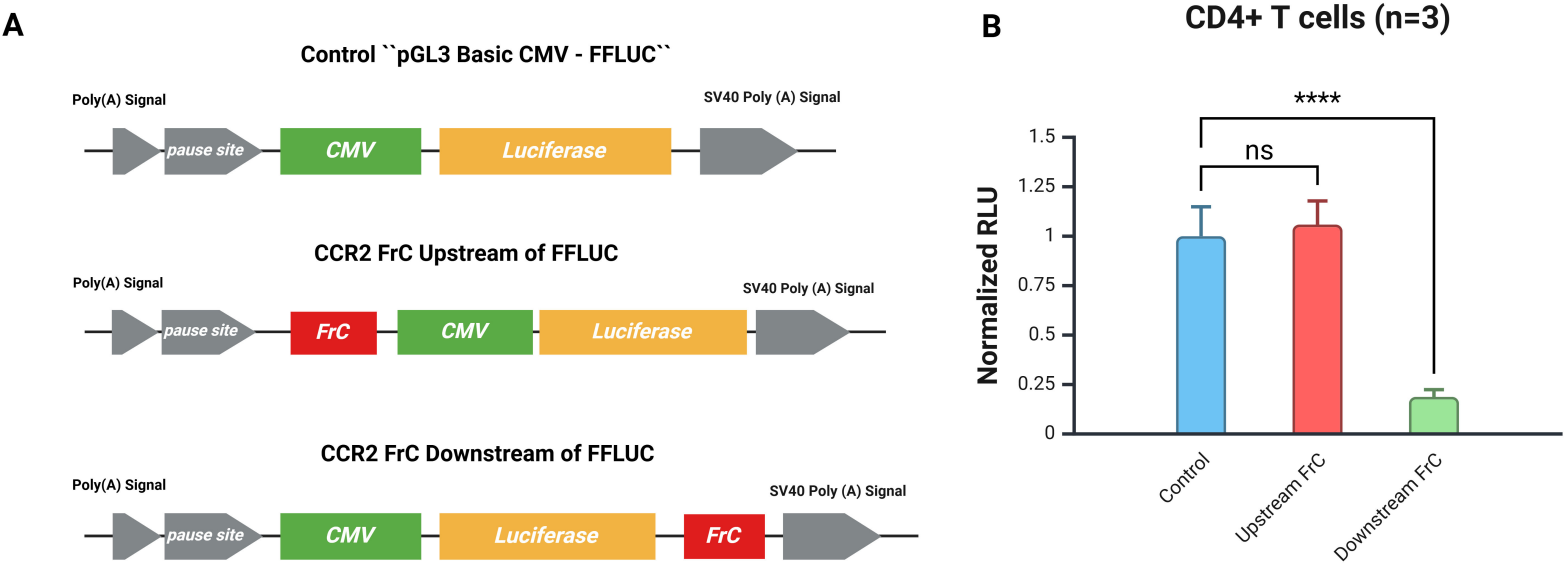
Positional cloning of FrC confirms that the effect is post-transcriptional. **(A)** Reporter plasmids are shown schematically. The CMV promoter that drives luciferase expression is the control plasmid (pGL3 Basic CMV-FFLUC); it lacks any CCR2 3`-UTR sequence. The FrC region of the CCR2 3’-UTR was cloned either upstream of the CMV promoter (CCR2 FrC Upstream of FFLUC) or downstream of the FFLUC sequence (CCR2 FrC Downstream of FFLUC). **(B)** Activated CD4+ T cells (n=3) transfected with CMV-GFP and the respective constructs. There was no significant difference (ns) in normalized RLU between the upstream FrC and control plasmids; however, the downstream FrC sequence significantly decreased luciferase activity when compared to the control plasmid (****p < 0.0001).

All the CCR2 3’-UTR subclones, termed FrA, FrB, FrC, FrD, and FrE, were then either PCR-amplified from FlUTR plasmid or cloned by restriction enzyme digestion using pCR-Blunt II-TOPO plasmid and pGL3-Basic-FFLUC reporter vector (**Supplementary Figure S1**). FrC fragment was also PCR-amplified, digested using unique restriction sites SmaI and XhoI, and the gel-isolated DNA product ligated just upstream of the CMV promoter using the same restriction sites to create clone Upstream FrC (**Figure 1A**). All recombinant plasmid clones were confirmed to be in the correct orientation by restriction enzyme digestion and DNA sequencing.

CD4+ T cells and macrophages were transfected using plasmids by electroporation and Lipofectamine 2000, respectively. To quantify luciferase activity, cells were incubated for 1–2 days post-transfection in culture. A portion of the cells was used to quantify RLU by luciferase luminometry. FFLUC assays were conducted in triplicate for each tested plasmid. Luciferase activity was measured using the Dual-Luciferase^®^ Reporter Assay System (Promega) on a plate reader, following the recommended procedure provided by the manufacturer. Cells were also co-transfected with pMaxGFP (Addgene), and transfection efficiency was normalized after a few days on a portion of the transfected cells, based on either epifluorescence microscopy or flow cytometry. Normalized RLU values are presented as mean ± SD.

### 2.5 Site-directed mutagenesis

Oligonucleotide primers were designed to produce three mutants of the 3’-UTR of CCR2: Deletion1, Mid-Deletion and Deletion-2. Deletion 1 is a 7 base pair or bp modification of the DNA sequence, Mid-deletion is a 14 bp modification of the DNA sequence, and Deletion 2 is 11 bp change of the DNA sequence.

**Supplementary Table S1** lists the three sets of DNA oligonucleotide primers used for site-directed mutagenesis. Standard PCR primer design guidelines were used to create the forward and reverse primers. Following amplification, DpnI (Roche) was added to the reannealed PCR products and incubated at 37 °C for 2 hours to selectively digest the methylated parental plasmid DNA, leaving the newly synthesized mutant strands intact. After DpnI digestion, agarose gel electrophoresis confirmed the correct size of the DNA band. Mutant plasmid DNA was transformed into E. coli and resultant ampicillin-resistant bacterial colonies were chosen at random and grown overnight at 37°C in LB supplemented with 100 μg/ml of ampicillin. DNA was isolated using a QIAprep Spin Columns (Qiagen, Germany), and successful incorporation of mutations was verified through Sanger DNA sequencing using primers flanking the mutation sites.

Plasmid DNA was isolated with the Spin Miniprep Kit (Qiagen, Germany), and the presence of intended mutations was confirmed via Sanger sequencing using primers positioned near the mutation sites.

### 2.6 GST and GST-hnRNPA0 fusion proteins

Plasmid encoding hnRNPA0 was obtained from Sino Biological (Cat# HG20649-U). The entire open reading frame was PCR-amplified, TOPO-cloned, and inserted into the GST fusion plasmid pGEX-5X-2 using SalI and NotI restriction enzyme sites (Addgene, originally from Pharmacia). Recombinant clones were verified as correct and in-frame by DNA sequencing.

A single GST-hnRNPA0 colony from a freshly streaked plate was inoculated into 3 ml of LB medium containing 100 µg/ml ampicillin and incubated at 37°C in a shaker. Following overnight incubation, 2.5 mL of the bacterial preculture was transferred into 250 mL of LB medium containing 100 µg/mL ampicillin. The culture was incubated at 37 °C with shaking at 250 RPM until the cell density reached an OD_600_ of 0.6. Subsequently, isopropyl β-D-1-thiogalactopyranoside (IPTG) was added to a final concentration of 1.0 mM to induce protein expression, and shaking incubation was continued for an additional 4 hours under the same conditions. Cells were harvested by centrifuging at 5000 RPM for 10 minutes at 4 °C, then resuspended in lysis buffer and disrupted by ultrasonication to extract the recombinant protein.

The GST and GST-fusion proteins were then purified using the ThermoFisher Pierce GST Spin Purification. Protein concentration was determined using the BCA assay, and the purified proteins were separated by SDS-PAGE followed by Coomassie Brilliant Blue staining and destaining. The results confirmed the expected sizes for GST and GST-hnRNPA0 proteins (**Supplementary Figure S4**). Purified proteins were cryopreserved at -20°C in small aqueous aliquots.

### 2.7 *In vitro* RNA transcription and EMSAs

To prepare RNA for electrophoretic mobility shift assays (EMSAs), 160 bp fragment C1 was PCR-amplified and cloned into the pCR-Blunt II-TOPO vector (Invitrogen). Plasmid was linearized with NotI, and RNA was transcribed *in vitro* using SP6 polymerase (NEB). Biotin-16-UTP (SIGMA-Aldrich) was included in the transcription reaction, following recommended procedures, and RNA was purified by using TRIzol. An irrelevant, mutant RNA was generated by introducing multiple A-to-G mutations in the ~160 bp C1 fragment and then cloned into pCR-Blunt II-TOPO vector. DNA sequence of the mutant clone confirmed, and RNA was similarly transcribed. RNA purity was confirmed by electrophoresing a small sample of it on a denaturing, 7M urea acrylamide gel and then staining the gel with EtBr.

Each biotin-labeled RNA was incubated for 30 min at RT with varying amounts of GST alone or GST-HnRNPA0 protein in a binding buffer (39). Bound RNA was then loaded on a 10% non-denaturing PAGE at 100V for 1–2 h in 0.5x TBE buffer (Thermo Scientifics). Gel contents were then transferred to a nylon membrane (Sigma) using the semi-dry transfer system (Bio-Rad) in pre-cooled 0.5x TBE buffer. After transfer, nylon membrane was incubated for 1 h at 37°C and subsequently overnight at 4°C in 5% blocking solution. On the following day, membrane was transferred into blocking solution and incubated with gentle shaking for 2 hours. Subsequently, it was treated at room temperature for 1 hr with streptavidin-conjugated horseradish peroxidase (ab7403, Abcam), diluted at 1:40,000 in 2.5% blocking buffer. The membrane was then washed multiple times using wash buffer (PBS, pH 7.5; 0.05% Tween-20). Finally, it was incubated for 5 minutes in detection buffer containing 100 mM Tris-HCl (pH 8.0) and 100 mM NaCl. Enhanced ECL reagent (BioRad) imaging of the membranes was then performed using the Cytiva Image Quant 8000 imaging System.

### 2.8 KO of 3`UTR of CCR2 via CRISPR-Cas9

To prepare ribonucleoprotein (RNP) complexes, sgRNAs shown in **Supplementary Table S1** and Cas9-GFP protein (from Integrated DNA Technologies) were mixed and incubated at room temperature for 20 minutes. CD4^+^ T cells were resuspended in T buffer (Invitrogen). A 10 µL volume containing 1 × 10^6^ cells was combined with the RNP mixture and electroporated using a Neon Electroporator set to 2000 V, 10 ms pulse width, and 3 pulses.

Following electroporation, cells were quickly transferred into 0.5 mL of pre-warmed RPMI 1640 medium containing fetal bovine serum (FBS) and incubated overnight at 37 °C in a humidified atmosphere with 5% CO. The next day, eGFP+ cells were positively sorted using Bigfoot Spectral Cell Sorter (ThermoFisher Scientific) and cultured for another 72 hr before further analysis. Following enrichment, eGFP-expressing CD4^+^ T cells were isolated via low-speed centrifugation, subjected to washing steps, and labeled CCR2 cell surface protein.

Protein levels were measured by FACS according to our previously published procedures (40). Cells were stained for CCR2 expression with anti-CCR2 antibodies for 1 hr in the dark at RT. We used anti-CD195-APC (clone J418F1; BioLegend), anti-CD192–APC or BV605 (CCR2; clone K036C2; BioLegend), anti-CD4–APC (clone: RPA-T4, BioLegend). DAPI solution was incorporated into the staining mix to avoid dead cells. Flow cytometric acquisition was performed on an LSRII instrument using FACSDiva, and data were analyzed using FlowJo software (v10.9.0).

### 2.9 Subcellular fractionation

Three days after positive cell sorting, the eGFP+ CD4+ T cells were harvested, and cytoplasmic and nuclear fractions of the cells were separated using the ThermoFisher Cell Fractionation Kit (Nuclear and Cytoplasmic Extraction Reagents, Thermo;78833). Briefly, cell pellets were resuspended in Cell Fractionation Buffer 1, and incubated on ice for 5 minutes. Following centrifugation at 500 × g for 5 minutes at 4 °C, the cytoplasmic supernatant was gently collected, while the nuclear pellet was resuspended in ice-cold nuclear extraction buffer. RNA from the cytoplasmic and nuclear fractions was then extracted using TRIzol. qRT-PCR for MALAT-1 and RPL30 were used to confirm acceptable subcellular fractionation of the nucleus and the cytoplasm, respectively.

### 2.10 mRNA stability quantification by treatment with alpha-amanitin

To measure mRNA stability, transcriptional inhibition was performed using alpha-amanitin (Sigma, Cat# A2263). Primary CD4^+^ T cells were first electroporated using CCR2 3’-UTR using CRISPR-Cas9-eGFP RNP complexes or mock controls, positively sorted for eGFP the next day, and then cultivated for 72 hr. Cells were then incubated with alpha-amanitin (10 µg/mL). Total RNA was isolated at post-treatment at time points of 0, 2, 4, 8, and 24 hours using the RNeasy Mini Kit (Qiagen). Quantitative RT-PCR was performed to assess CCR2 mRNA decay over time, and UBC (Ubiquitin C) was used as a housekeeping control due to its known half-life stability (~2.5 hours). The relative mRNA abundance at each time point was normalized to time zero using the ΔΔCt method. RNA half-life were calculated using standard curves, and comparisons between KO and control conditions were used to determine the effects of the CCR2 3’-UTR on mRNA half-life.

### 2.11 Quantitative reverse transcription PCR

Total RNA was isolated from a portion of the cell population using the RNeasy Kit (Qiagen) following the manufacturer’s protocol, while the remaining cells were stained to assess CCR2 surface expression. cDNA was generated using Invitrogen’s One-Step RT-PCR System and analyzed via qPCR on a Bio-Rad CFX96 instrument with the SuperScript III Platinum SYBR Green (Invitrogen). Fold change differences were calculated by the ΔΔCt method. The complete list of qRT-PCR primers is provided in **Supplementary Table S1**.

### 2.12 Single-cycle HIV production and T cell transduction

HIV-cycT1-IRES-YFP (HIV-CIY) along with pSM-ADA Env, pSRα-YU2 Env, or the pan-tropic control plasmid pME-VSV-G was introduced into 293T cells at approximately 70% confluency using the calcium phosphate transfection method. Seventy-two hr later, pseudotyped lentiviral particles were harvested from the supernatant of the cultured cells. For transduction assays, GHOST-HI5 cells and GHOST.CCR2B cells were used as target cells. Transduction efficiency was quantified by flow cytometry. The presence of CCR2 and absence of CCR5 on GHOST.CCR2B cells were confirmed via FACS analysis. Following transduction with the pseudotyped HIV particles, eYFP-positive cells were analyzed by flow cytometry 72 hr later.

### 2.13 Statistical analyses

Statistical analyses were performed using GraphPad Prism version 9.0 (GraphPad Software, La Jolla, CA, USA) on a Windows operating system. Comparisons between groups were evaluated using a two-tailed Student’s t-test. Significance levels were marked as follows: * p < 0.05; ** p < 0.01; *** p < 0.001; and **** p < 0.0001.

## 3 Results

### 3.1 The 3’-UTR of CCR2 is inhibitory to gene expression

**Figure 2:**
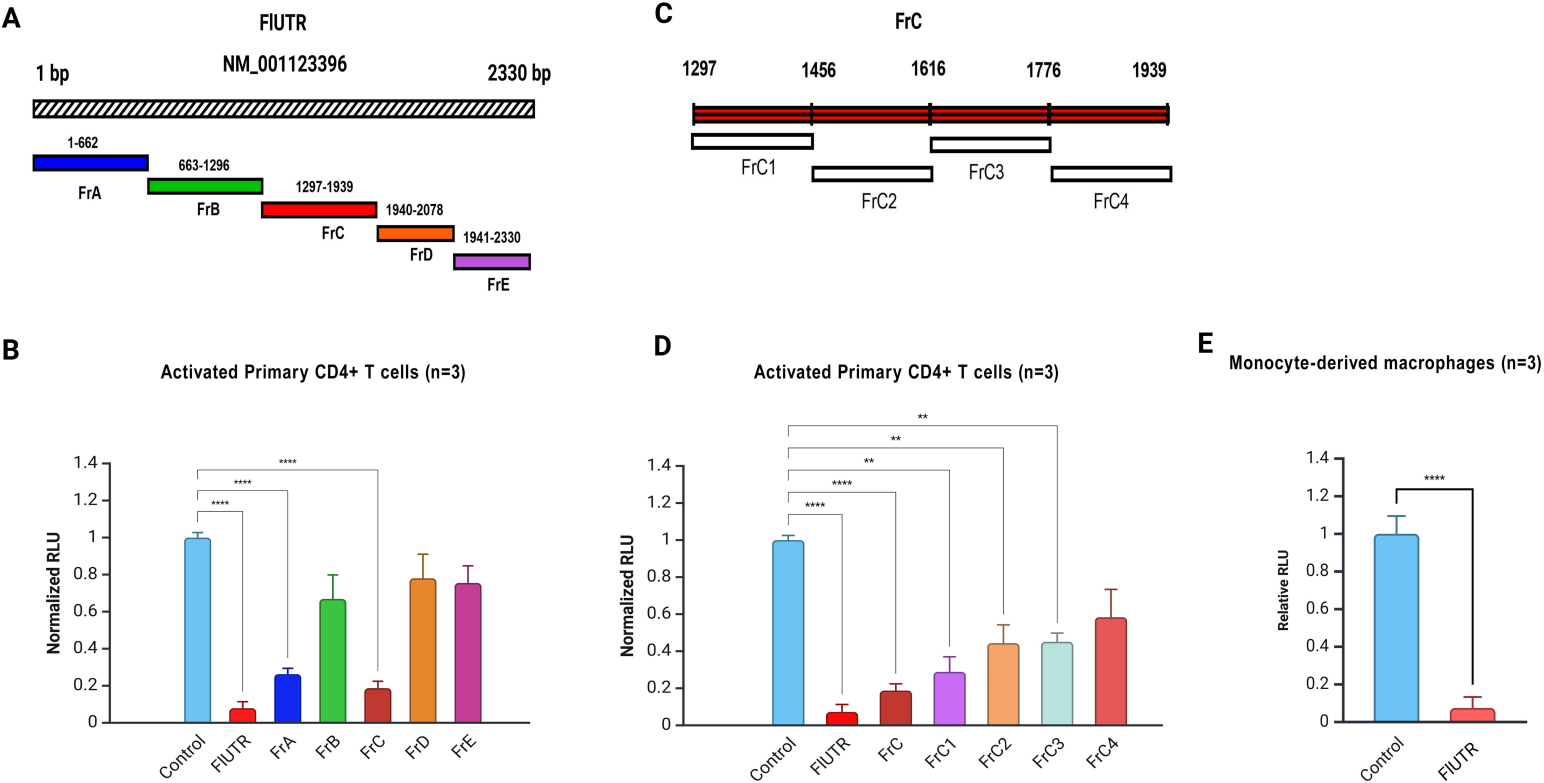
CCR2 3’-UTR fragments regulate gene expression in primary CD4+ T cells and macrophages. **(A)** Schematic representation of the full-length CCR2 3’-UTR (2330 bp) and its fragments (FrA-FrE). **(B)** Normalized relative luciferase units (RLU) in activated primary CD4+ T cells electroporated with the full-length CCR2 3’-UTR (FlUTR) or its fragments (FrA-FrE), each directionally cloned immediately downstream of CMV-FFLUC reporter. Significant inhibition of FFLUC activity was observed in cells transfected with FlUTR, FrA, and FrC. **(C)** Detailed schematic of Fragment C (FrC, 641 bp), which was further sub-divided into fragments FrC1-FrC4 for finer analysis; each was cloned just downstream of CMV-FFLUC reporter. **(D)** Normalized RLU in activated primary CD4+ T cells transfected with FrC or FrC1-FrC4. Among the subclones, FrC1 exhibited the greatest inhibition of FFLUC activity. **(E)** Normalized RLU in primary human macrophages derived from monocytes transfected with full-length CCR2 3’-UTR. Data represent the mean ± SD from three independent experiments using cells from three different donors. Luciferase assays were performed in triplicate for each condition. Statistical significance was determined using one-way ANOVA followed by Bonferroni’s multiple comparison test or a two-tailed unpaired t-test (*p < 0.05, **p < 0.01, ****p < 0.0001).

All plasmids containing sequence of the CCR2 3′-UTR were cloned by inserting them downstream of a CMV-driven firefly luciferase (FFLUC) gene to evaluate post-transcriptional regulatory effects (**Figure 2**). These plasmids were transfected into primary, activated human CD4+ T cells by electroporation and monocyte-derived macrophages using cationic lipids. A GFP reporter plasmid was co-transfected as a normalization control to assess transfection efficiency. As a positive control, we used a CMV-FFLUC reporter construct that did not contain any CCR2 3′-UTR sequences. Cells were incubated 1–2 d post-transfection, lysed, and FFLUC activity was measured using a luminometer and expressed in normalized relative luminescence units (RLU).

Full-length CCR2 3’-UTR (Fl-UTR, 2330 bp) significantly reduced FFLUC expression by approximately 13-fold in primary CD4+ T cells (p<0.0001; **Figures 2B, C**). Similarly, the 630 bp fragment (FrA) and 641 bp fragment (FrC) reduced RLU by 5- and 6-fold, respectively (p<0.0001; **Figure 2B**). Other regions of the CCR2 3’-UTR also demonstrated varying levels of suppression in gene expression (**Figures 2B–D**). In primary human macrophages derived from monocytes, the full-length CCR2 3’-UTR reduced reporter gene expression by approximately ~20-fold (p<0.001; **Figure 2E**). Based on these findings, we focused on and further investigated the most inhibitory fragments, the 640 bp FrC and its subclone, the 160 bp FrC1 DNA fragment (**Figures 2A–C**).

### 3.2 Post-transcriptional regulation, focused on the FrC region

To determine whether the inhibitory effect of the full-length CCR2 3′-UTR occurs at the post-transcriptional level, the FrC fragment was inserted upstream (5′) of the CMV promoter (**Figure 1A**). This upstream cloning did not alter FFLUC activity compared to the control (**Figure 1B**). These findings are consistent with the fact that the CCR2 3′-UTR modulates gene expression at the post-transcriptional level.

### 3.3 160 bp FrC1 region and RNA-binding proteins

**Figure 3:**
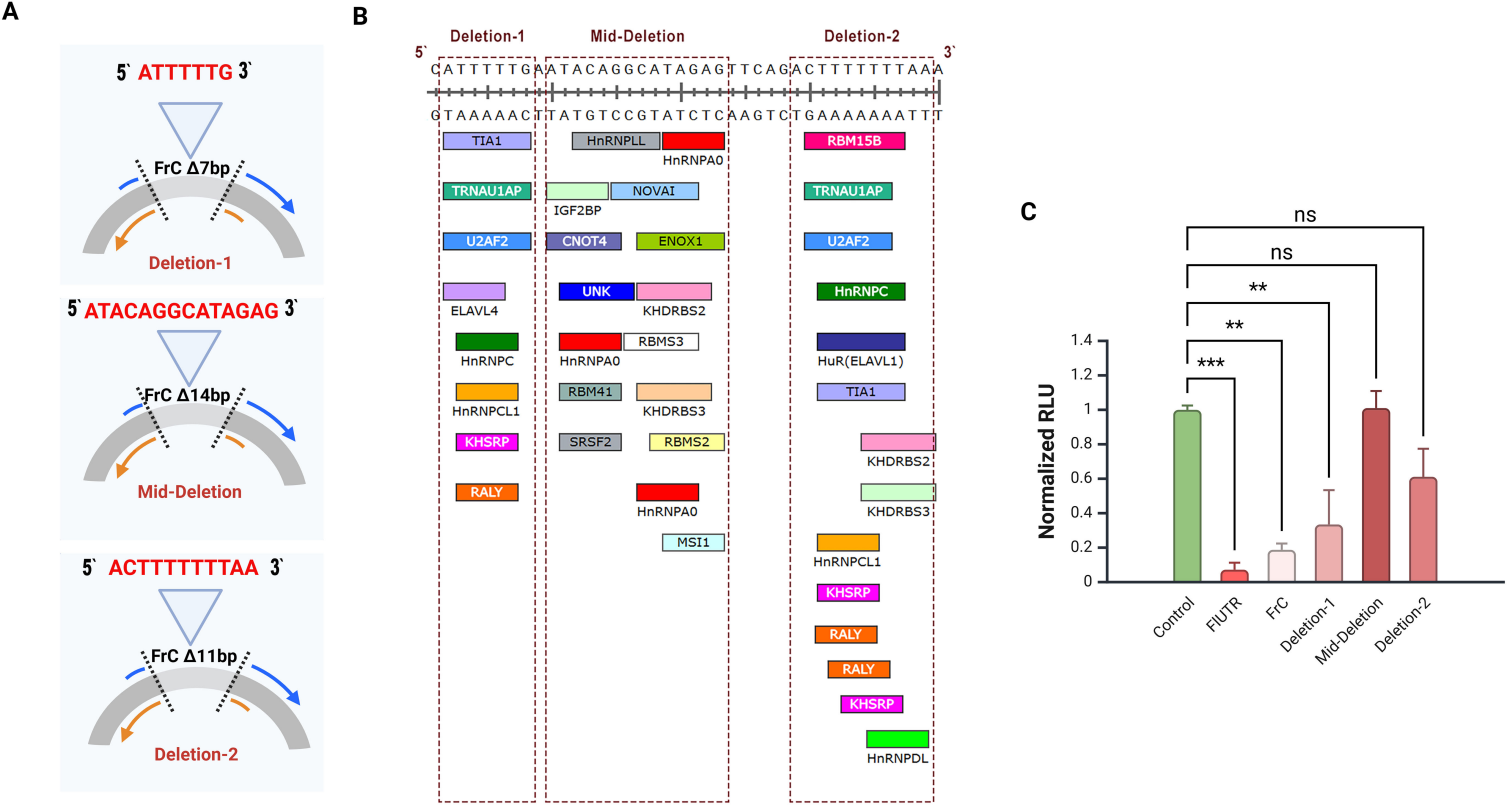
Site-directed mutagenesis of the CCR2 3’-UTR FrC region. **(A)** Schematic representation of the three deletion constructs of the FrC region of the CCR2 3’-UTR created by site-directed mutagenesis. 7 bp (5’-ATTTTTG-3’) was removed in Deletion-1, 14 bp (5’-ATACAGGCATAGAG-3’) in the Mid-Deletion, and 11 bp (5’-ACTTTTTTTAA-3’) in Deletion-2. DNA deletions were confirmed by Sanger sequencing. **(B)** Nucleotide sequences of predictive RBPs on FrC1. Shown are RBPs located in the 1417–1456 bp region of the CCR2 3’-UTR. The three deletions constructed using sitedirected mutagenesis are indicated by the red rectangles **(C)** Primary CD4+ T cells electroporated with CMV-GFP and each of the deletion constructs, full-length CCR2 3’-UTR (FlUTR), or FrC; normalized luciferase activity (RLU) is shown. When compared to wild-type FrC, the Mid-Deletion and Deletion-2 constructs eliminated the inhibitory effect of FrC. **p < 0.01, ***p < 0.001. ns, not significant.

The 640 bp FrC region and its 160 bp subclone (FrC1) were analyzed for miRNA and RBP binding sites. Biocomputational analysis identified dense clusters of potential binding sites in small sequences (7–14 bp) within FrC1.To validate the importance of these sequences, site-directed mutagenesis was employed to generate the following constructs: Deletion1 (7 bp), Mid-deletion (14 bp), and Deletion2 (11 bp) (**Figures 3A, B**).

RLU activity of the Mid-deletion and Deletion-2 mutants revealed that the absence of these regions eliminated the inhibitory effect of FrC, suggesting that those nucleotide sequences harbor one or more critical regulatory elements (**Figure 3C**). To analyze predictive RNA binding regions, the RBPmap tool developed by Paz et al. was used (https://rbpmap.technion.ac.il/) (41). Predictive analysis identified three binding sites within the mid-deletion region for the RBP hnRNPA0, indicating its potential functional role as an inhibitor in this regulatory mechanism.

In addition, biocomputational analysis of the Deletion-2 region identified multiple AU-rich elements predicted to function as binding sites for RBPs. To evaluate the significance of this region, KO experiments were performed targeting RBPs with multiple binding sites, including KHSRP, hnRNPDL and RALY, to assess their impact on CCR2 expression. KO of these RBPs did not result in a significant change in CCR2 protein expression (**Supplementary Figure S3**). We then turned to hnRNPA0 and its effects on CCR2 post-transcriptional regulation.

### 3.4 hnRNPA0 binding to RNA

**Figure 4:**
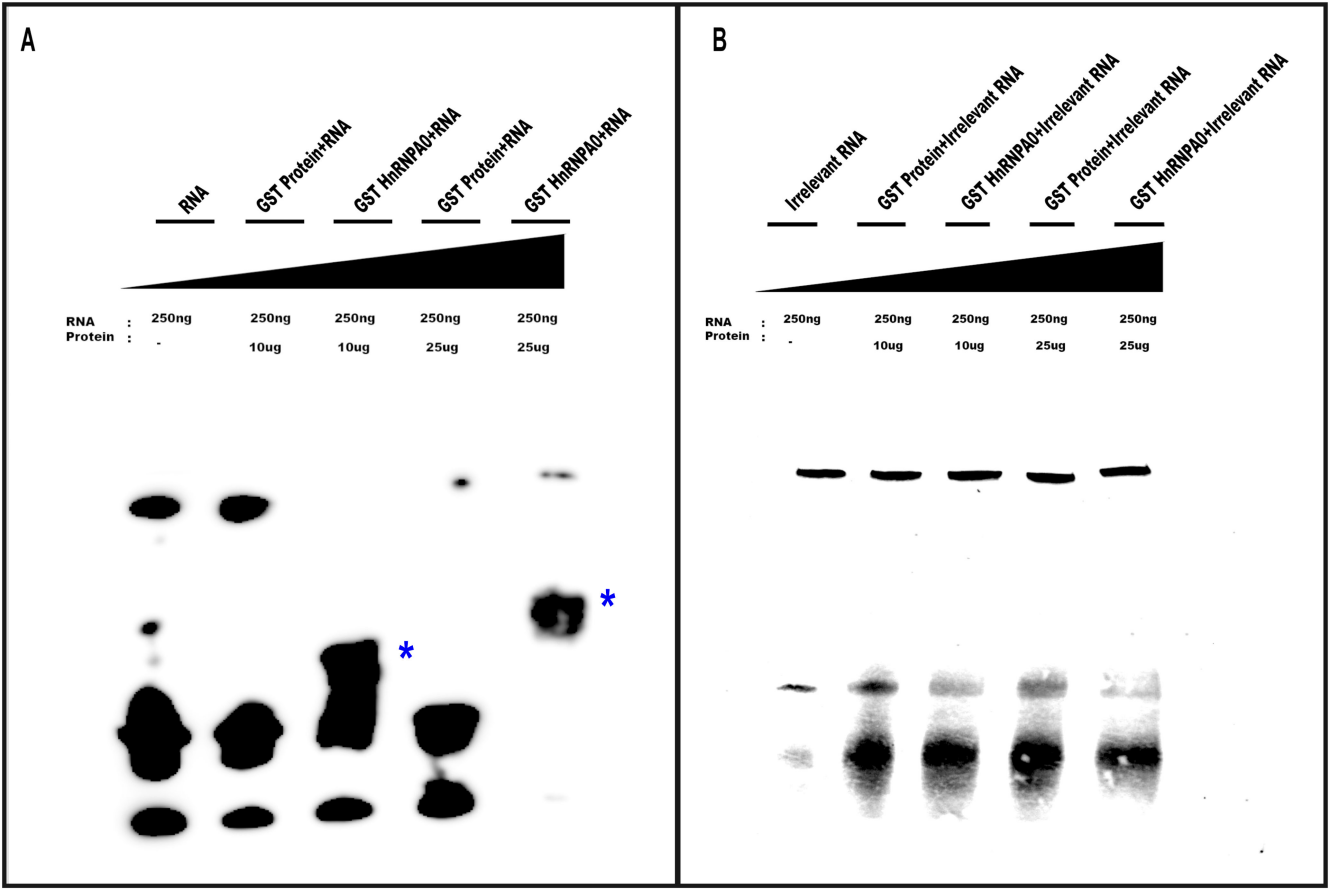
EMSAs demonstrating specific binding of GST-hnRNPA0 to the FrC1 region of CCR2 3’-UTR. **(A)** EMSA results using 250 ng of biotin-UTP-labeled RNA derived from the FrC1 region of CCR2 3’-UTR and varying amounts of GST-hnRNPA0 fusion protein (10 μg and 25 μg). A distinct electrophoretic shift was observed with FrC1 RNA in the presence of GST-hnRNPA0, indicating specific binding (bands labeled with *). GST alone did not show any shift, confirming the specificity of the interaction. **(B)** Control EMSA using irrelevant mutant RNA lacking AU-rich elements (ARE). No shift of the RNA was observed with either GST-hnRNPA0 or GST alone.

To confirm hnRNPA0 binding to the FrC1 region, a GST-hnRNPA0 fusion plasmid construct was made. The entire coding sequence of hnRNPA0 was PCR-amplified, cloned into a GST expression vector, and DNA-sequenced. The construct was transformed into *E. coli*, grown exponentially, treated with IPTG, and resulting cell pellets were lysed using ultrasonication. Cell lysates were run over glutathione affinity columns and purity of resultant GST proteins and GST-hnRNPA0 fusion proteins verified by SDS-PAGE followed by Coomassie staining (**Supplementary Figure S4**). The 160 bp FrC1 RNA fragment was obtained by *in vitro* transcription using SP6 polymerase enzyme and biotin-UTP. As a control, a mutant RNA lacking ARE regions was similarly synthesized. EMSAs using these RNAs and GST-hnRNPA0 protein demonstrated a clear shift for the FrC1 RNA, confirming hnRNPA0 binding (**Figure 4A**). No shift was observed when GST alone (**Figure 4A**) or when the mutant RNA was used (**Figure 4B**).

### 3.5 Effect of CCR2 3’-UTR on gene and protein expression

**Figure 5:**
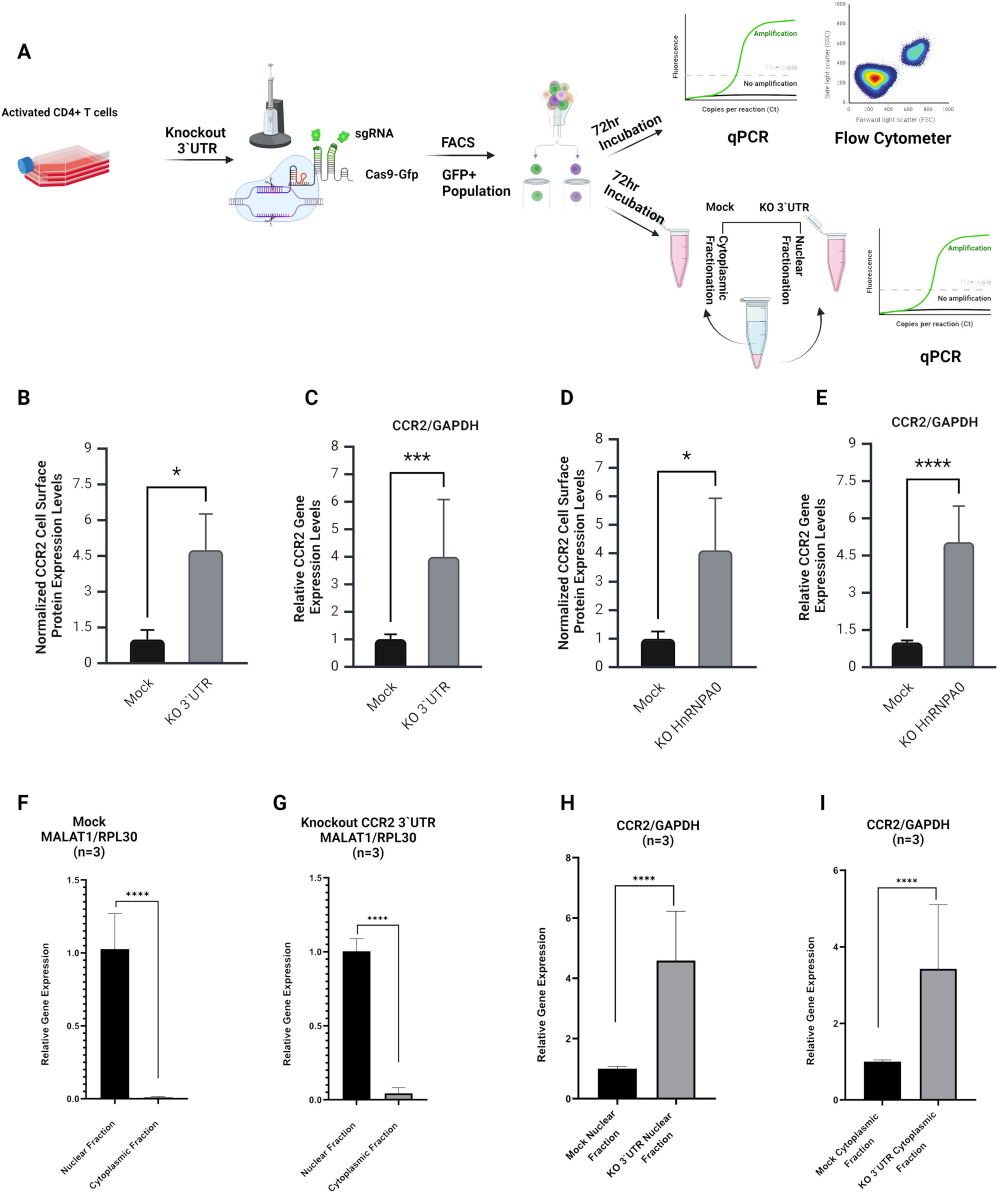
Analysis of CCR2 3’-UTR and hnRNPA0 knockout effects on CCR2 expression and RNA sub-cellular fractionation. **(A)** Schematic representation of the experimental workflow. Primary CD4+ T cells were activated and electroporated using Cas9-GFP and sgRNAs targeting the entire CCR2 3’-UTR. GFP+ cells were sorted by FACS and incubated for 72 hours prior to RNA and protein analyses. The same experimental setup was also used for qPCR analyses following cell fractionation to assess nuclear and cytoplasmic transcript distributions. **(B, C)** Flow cytometry and qPCR results for CCR2 3’-UTR KO cells, showing a significant increase in cell surface CCR2 expression (~4.5-fold; p < 0.05) and CCR2 mRNA levels (~4-fold; p < 0.001) compared to mock transfection, which did not include any gRNAs. **(D, E)** Effects of hnRNPA0 KO on CCR2 gene expression. Flow cytometry and qPCR revealed a significant increase in CCR2 protein (~4-fold; p < 0.05) and mRNA (~4.7-fold; p < 0.001) expression compared to mock-treated cells. **(F, G)** Nuclear and cytoplasmic RNA fractionation. qPCR analysis of MALAT1 (nuclear marker) and RPL30 (cytoplasmic marker) confirmed successful sub-cellular fractionation. **(H, I)** In CCR2 3’-UTR KO cells, an increase of CCR2 mRNA was observed in both nuclear and cytoplasmic fractions (p < 0.0001). Data represent mean ± SD from three independent experiments (n=3). Statistical analysis was performed using a two-tailed unpaired t-test or one-way ANOVA with Bonferroni correction for multiple comparisons. *p < 0.05; *** p< 0.001, ****p < 0.0001.

To evaluate the effect of the full-length CCR2 3’-UTR on CCR2 gene expression, CRISPR-Cas9 KO experiments were performed in activated, human primary CD4+ T cells. Cells were electroporated with Cas9-GFP and two sgRNAs targeting the 5’ and 3’ termini of the CCR2 3’-UTR (**Figure 5A**). One day after cell electroporation, GFP+ cells were sorted by FACS and cultured for 72 h. qPCR analysis showed a ~4-fold increase in CCR2 mRNA expression, while flow cytometry showed a ~4.5-fold increase in CCR2 protein levels (p<0.001; **Figures 5B, C**). These results confirm that the CCR2 3’-UTR down-modulates both CCR2 RNA and protein.

### 3.6 Role of RBPs and hnRNPA0 in CCR2 regulation

The effects of RBPs hnRNPA0, hnRNPDL, RALY, and KHSRP on CCR2 regulation were assessed using CRISPR-Cas9 KO. Knockout efficiency of CCR2 3′-UTR and RBP genes was confirmed by nested PCR and qPCR, respectively (**Supplementary Figure S5**). Genes were KO’d using sgRNAs, and the resulting changes in CCR2 expression were examined. Among them, hnRNPA0 knockout caused a strong increase in CCR2, with mRNA levels rising by ~4.7-fold and surface protein by ~4-fold (**Figures 5D,E**). However, CCR2 gene expression remained largely unchanged following the knockout of RALY, hnRNPDL and KHSRP. (**Supplementary Figure S3**).

### 3.7 Cell fractionations and comparison of transcript levels

To investigate the mechanisms underlying CCR2 regulation by its 3’-UTR, after KO of the 3’-UTR subcellular fractions were isolated and tested by qPCR. Successful subcellular fractionation was confirmed using MALAT1 and RPL30 as specific nuclear and cytoplasmic markers (**Figures 5F, G**). In the case of CCR2, both nuclear and cytoplasmic RNA levels were significantly increased in the absence of its 3’-UTR, suggesting that 3’-UTR KO stabilizes CCR2 mRNA, leading to enhanced translation (**Figures 5H, I**).

### 3.8 Role of CCR2 in HIV infection

**Figure 6:**
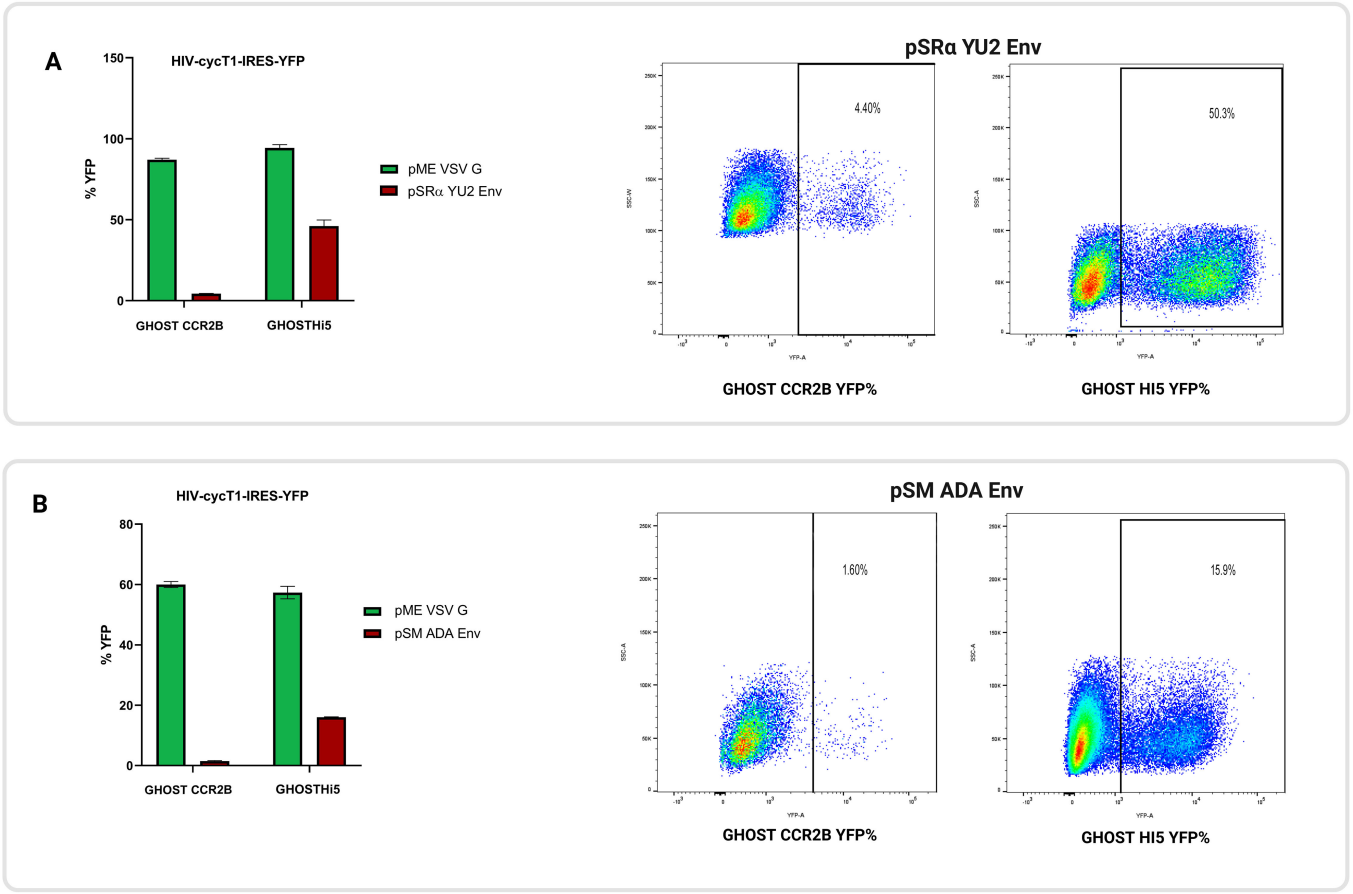
CCR2 functions as an HIV co-receptor. Replication-defective HIV encoding eYFP were pseudotyped with VSV-G or R5-tropic Env (either YU2 or ADA) and used to infect GHOST.CCR2B and GHOST.Hi5 cells. To determine rates of transduction, cells were analyzed by flow cytometry. **(A)** GHOST.CCR2B cells showed 4.4% YFP+ cells when R5-tropic YU2 Env was used, whereas GHOST.Hi5 cells showed 50.3% YFP+ cells. **(B)** GHOST.CCR2B cells had 1.6% YFP+ cells when R5-tropic ADA Env was used, whereas GHOST.Hi5 cells had 15.9% eYFP+ cells. The means ± SD of three separate experiments are shown.

Given the potential therapeutic importance of CCR2 in HIV treatment and its amino acid homology to CCR5, GHOST.CCR2B cells were infected with a replication-defective HIV vector pseudotyped with YU2 or ADA R5-tropic Env or VSV-G. Flow cytometric analysis revealed that those cells were susceptible to R5-tropic Env-mediated transduction, at roughly ~10% of the level of GHOST.Hi5 cells (**Figure 6**). Additionally, those cells were confirmed by flow cytometry to express CCR2 on the cell surface and not CCR5 (**Supplementary Figure S2**).

### 3.9 Increased CCR2 mRNA half-life following 3′-UTR knockout

**Figure 7:**
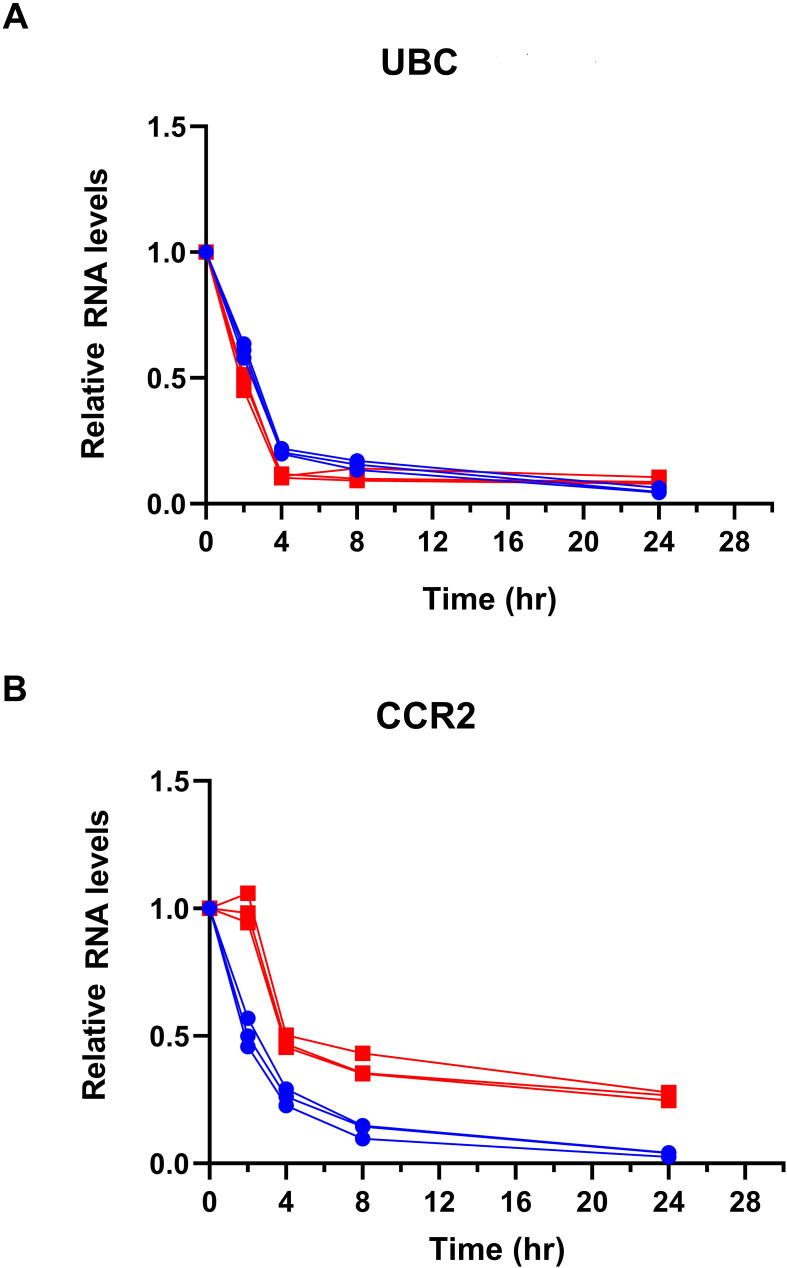
CCR2 3′-UTR KO increases CCR2 mRNA stability following transcriptional inhibition. Primary human CD4^+^ T cells were electroporated with Cas9-GFP and sgRNAs targeting the CCR2 3′-UTR or mock control. Red: KO of CCR2 3’-UTR; Blue: mock KO of CCR2 3’-UTR. After 72 hours, cells were treated with 10 µg/mL alpha-amanitin to block RNA polII transcription. RNA was collected at 0, 2, 4, 8, and 24 hours, and CCR2 expression was measured by qRT-PCR, normalized to the housekeeping gene UBC. **(A)** UBC RNA levels. **(B)** CCR2 RNA levels. Data are shown as mean ± SD from three independent experiments.

To directly test the effect of the CCR2 3′-UTR in mRNA stability, we treated cells with α-amanitin to measure mRNA decay kinetics. Primary CD4+ T cells after mock or KO of the CCR2 3′-UTR were treated with 10 µg/mL α-amanitin, and mRNA levels were quantified by qRT-PCR at 0, 2, 4, 8, and 24 h after α-amanitin exposure. UBC, a housekeeping gene with a known half-life of roughly 2.6 hours, was used as an internal control. The half-life of CCR2 transcripts increased from 2.51 hr in control cells to 3.58 hr in 3′-UTR KO cells, while UBC mRNA decay remained largely unchanged under these conditions (2.59 hr vs. 2.35 hr) (**Figure 7**). This indicates that KO of the CCR2 3′-UTR results in enhanced mRNA stability by ~43%, suggesting that the 3′-UTR of CCR2 reduces its RNA half-life under normal cellular conditions.

## 4 Discussion

Although CCR2 is widely recognized for its roles in immune cell movement, inflammation, and possibly HIV disease, the specific molecular mechanisms controlling its gene expression are not fully understood. We examined the effect of the CCR2 3’-UTR on gene expression through post-transcriptional mechanisms in this study. It has been previously reported that 3’-UTRs play a critical role in controlling mRNA regulation, including stability, localization, and translation. Post-transcriptional regulation of gene expression by the 3’-UTR is essential during development and in the pathogenesis of diseases (42–44). The regulatory activity of 3’-UTRs depends on cis-RNA elements and trans-acting factors. AU-rich element (ARE) motifs are found in approximately 5-8% of protein-coding genes and can increase or decrease gene expression, depending on RNA-binding proteins (45–47).

A key function of CCR2 in inflammation involves recruiting monocytes and other components of the immune system to areas of tissue injury or infection, mainly via binding to its ligand CCL2 (monocyte chemotactic protein-1, MCP-1) (3). This process forms the cornerstone of both acute and chronic inflammatory responses. One of the most critical functions of CCR2 is its involvement in controlling microbial infections and tissue repair; however, it is also associated with pathological conditions such as atherosclerosis, fibrosis, and cancer (48, 49). CCR2 is therefore of great importance as both a critical target in modulating the immune system and a potential therapeutic approach for treating inflammation-associated diseases.

CCR2 is expressed as different transcript variants, including a shorter form (CCR2a) with a truncated 3′-UTR and a longer CCR2b isoform, which predominates in immune cells. In our study, only the ~2.3kb 3`UTR variant was detected across multiple blood donors. The functional 3′-UTR region we investigated (FrC) is present in both isoforms, but our data primarily reflect the regulatory role of the extended 3′-UTR in the CCR2b transcriptOur findings demonstrate that the full-length 3′-UTR of CCR2 strongly suppresses CCR2 gene expression in both primary CD4^+^ T cells and macrophages. Results of the FFLUC reporter assays suggest that the full-length 3’-UTR is markedly inhibitory role since it reduces reporter gene expression by approximately 13-fold in CD4+ T cells. Shorter 3’-UTR fragments, FrC and FrC1, which are 641 and 160 bp in length respectively and have specific binding sites for RBPs, were both very inhibitory to CCR2 gene expression. These findings support previous studies showing that 3’-UTRs have the potential to control mRNA stability and translation efficiency (42, 50, 51). Our positional cloning analyses showed that the 640 bp FrC region regulates gene expression post-transcriptionally; the nucleotide fragment had to transcribed and be part of the RNA to be inhibitory.

Regulation of gene expression at the post-transcriptional level is essential for maintaining cellular homeostasis and is implicated in the pathogenesis of various human diseases (52). The roles of RBPs and microRNAs on mRNA stability, translation, and cellular location are crucial as fundamental mechanisms in regulating gene expression. Instability in these processes, however, leads to various pathological conditions, including cancer (53, 54), neurodegenerative diseases (55), and immune disorders (56). Post-transcriptional regulation of genes is critical to improved understanding of disease mechanisms and development of therapeutic interventions (57). In particular, RNA targeting strategies and modifications have been suggested to be promising in the field of precision medicine (58–60).

One of the most important findings here is that the 3’-UTR plays a critical role in regulating CCR2 gene expression, specifically by interacting with hnRNPA0, which, based upon bioinformatics, contains >20 different binding sites for hnRNPA0 on FrC and 3 different binding sites on FrC1. Using site-directed mutagenesis, it was determined that the inhibitory effect in the reporter assay was eliminated by removing all 3 hnRNPA0 binding sites within the FrC1 fragment. Eliminating hnRNPA0 using CRISPR-Cas9 and gRNAs significantly elevated mRNA levels as well as cell surface protein expression. These results align with previous studies identifying hnRNPA0 as a key regulator of mRNA stability for proinflammatory cytokines (37, 38). hnRNPA0 has previously been reported to play a crucial role in the post-transcriptional control of inflammatory genes such as TNF-α and IL-6 (38). Its phosphorylation by MK2 (MAPKAP-K2) is critical for regulating mRNA stability (37, 61). Our study supports this mechanism, and we hypothesize that the 3`-UTR of CCR2 binds one or more RBPs, including hnRNPA0, and retains the RNA in the nucleus so it cannot be translated in the cytoplasm. This result parallels data showing that hnRNPA0 regulates mRNA stability in the context of inflammation and cancer (62–64). In particular, it emphasizes the important role of AREs and the RBPs in resistance to chemotherapy and in translational regulation of RNAs encoding proinflammatory cytokines (65).

Our study shows that hnRNPA0 deficiency may cause immune cells to shift to a pro-inflammatory state by increasing CCR2 mRNA levels. This suggests that hnRNPA0 may be a central regulator that controls not only CCR2 gene expression but a broader inflammatory gene network, suggesting a greater need to understand its role in diseases in this context. Editing 3′-UTR sequences and modulating mRNA stability is a powerful tool for controlling gene expression dynamics. 3′-UTRs contain critical elements determining mRNA stability and degradation.

mRNA half-life assays using α-amanitin further validated the regulatory role of the CCR2 3′-UTR. The observed increase in CCR2 mRNA half-life following removal of its 3′-UTR suggests that this region promotes transcript destabilization, likely through recruitment of RNA-binding proteins or other regulatory elements. Notably, the unchanged half-life of the control UBC gene confirms the specificity of this effect. These results underscore the importance of the CCR2 3′-UTR in fine-tuning transcript longevity and strengthen our conclusion that post-transcriptional mechanisms, particularly those involving the 3′-UTR, are critical for CCR2 expression control.

The 3′-UTR plays a role in controlling mRNA degradation, thereby affecting the overall transcript levels maintained in the cell (66). This mechanism regulates gene expression dynamics in both natural and synthetic biological circuits (66). In particular, regulation of the number, placement, and type of regions such as AREs provides important control over mRNA stability. Here we show significant increases in CCR2 gene and protein expression when either the entire 3′-UTR or hnRNPA0 is KO’d. This suggests that hnRNPA0 is a balancing factor in properly functioning inflammatory processes. hnRNPA0 deficiency might disrupt checkpoints in the DNA repair mechanism, increasing sensitivity to chemotherapy in p53-mutant cells (67). This mechanism represents a shift from transcriptional to post-transcriptional regulation and suggests that hnRNPA0 functions as a “successor” regulator in the presence of p53 deficiency (67). These findings suggest that hnRNPA0 is a key contributor to chemotherapy resistance and underscore its potential as a promising target for therapeutic interventions. Thus, it is quite likely that hnRNPA0 plays a regulatory role in controlling inflammatory gene expression during stress responses (67, 68).

Variants in some 3`-UTRs have been reported as significant in cancer pathogenesis. In one study, 3`-UTR mutations had an impact on mRNA stability and translation efficiency in prostate cancer patients (69), highlighting the critical regulatory effect of these regions in cancer biology. 3`-UTR mutations in genes such as IGF1R, MSI2, MBD2, and ASCL1 have been shown to contribute to oncogenic processes by increasing mRNA stability or translation. In KO studies, these mutations in the 3`-UTR have been shown to significantly increase protein expression, even in heterozygous cases (69). Specific RBPs, including hnRNPA0, effect the expression of inflammatory genes by binding to AREs, playing a key role in controlling immune responses.

Here, we observed that KO of CCR2 3’-UTR caused an increase in both nuclear and cytoplasmic RNA transcript levels, showing that the 3’-UTR plays an essential regulatory role in the intracellular distribution of mRNA. Its deficiency disrupts this balance, causing transcript accumulation in both sub-cellular compartments. This effect may be due to changes in RNA stability. hnRNPA0 deficiency causes a rise in infectious HIV particle production by increasing the transport and translation of viral RNA from the nucleus to the cytoplasm, suggesting that hnRNPs play a vital role in nucleic acid transport from the nucleus to the cytoplasm within the cell (70). It has been suggested that hnRNPA0 may bind to the HIV promoter and regulate transcription, but mechanistic details have yet to be elucidated (70). From a broader perspective, it is clear that hnRNPA0 should be considered an important research target in understanding the molecular mechanisms of inflammatory diseases and other immune-related pathologies. Considering the contribution of this increase in CCR2 transcripts to translation, stability may also impact RNA translation.

Our nuclear and cytoplasmic sub-cellular fractionation experiments revealed that CCR2 RNA significantly increased in both the nucleus and cytosol after KO of its 3’-UTR. This result suggests that the CCR2 3’-UTR functions as a regulatory element controlling the amount and distribution of RNA transcripts. These findings suggest that 3′-UTR-mediated regulation of CCR2 mRNA may not be limited to nuclear retention. Although we initially hypothesized that hnRNPA0 binding contributes to transcript destabilization through nuclear sequestration, the observed increase in CCR2 mRNA in both nuclear and cytoplasmic fractions following 3′-UTR deletion implies broader regulatory roles. Notably, hnRNPA0 has been shown to be stabilized and activated in the cytoplasm via MAPKAP-K2-mediated phosphorylation (71) supporting the idea that it may also participate in cytoplasmic mRNA decay. Together, these observations indicate that hnRNPA0 may influence CCR2 mRNA turnover in both nuclear and cytoplasmic compartments.

. miRNAs binding to the 3’-UTR may play a similar role, but as of yet we have not obtained any direct evidence of that occurring for CCR2.

3’-UTRs have been reported to play essential roles in various processes, such as mRNA nuclear transport, stability, and translation efficiency (72). For example, RNA-binding proteins bind to 3’-UTRs, leading to RNA transport to the cytoplasm or translational repression (73–75). Within this context, KO of the CCR2 3’-UTR may contribute to RNA accumulation in the nucleus and the cytoplasm by enhancing mRNA stability. The increase in nuclear RNA levels highlights a potential negative effect on the tightly regulated transport of CCR2 mRNA, leading to an unbalanced accumulation of inflammatory gene transcripts. We hypothesize that RNAs that accumulate in the nucleus are degraded. We suggest that 3’-UTRs play a dual regulatory role in mRNA stability and subcellular transport. The accumulation of CCR2 transcripts in the nucleus and cytoplasm indicate that this 3’-UTR region is critical for regulating translation of RNA. These findings parallel studies in which RBPs and 3’-UTRs control the pre-translational distribution of transcripts (76–78).

As members of the chemokine receptor family, both CCR2 and CCR5 play essential roles in migrating immune cells into the tissue (79, 80). While it is well-known that CCR5 act as the main co-receptor for R5-tropic HIV infection (81, 82), the coding sequence of CCR2 is more than 70% homologous to that of CCR5, highlighting the potential contribution of this protein to HIV binding and entry into immune cells. Although prior work provided limited support for the direct involvement of CCR2 in the replicative cycle of R5-tropic HIV, CCR2 may assist in the migration of immune cells to target tissues (83). Although CCR2 is not a commonly used entry co-receptor for HIV *in vivo*, rare strains have been reported to utilize CCR2, and its primary role in HIV pathogenesis is linked to monocyte recruitment and inflammation, which may facilitate infection by attracting new target cells to infected tissues (23, 84).

Here we show that CCR2 can function as co-receptor for R5-tropic HIV, but only at ~10% of the level of CCR5. We readily admit, however, that there is no direct evidence that CCR2 functions as an HIV co-receptor in humans.

Our study also demonstrates the role of RBPs, particularly hnRNPA0, in the post-transcriptional regulation of the CCR2 3’-UTR, indicating that could impact the spread of HIV. Controlling CCR2 gene expression may slow the spread of HIV in the body by inhibiting the migration of immune cells to sites of inflammation, where the virus is actively replicating. In particular, reducing CCR2 expression by targeting the binding of hnRNPA0 to CCR2 RNA may offer a new therapeutic strategy aimed at controlling R5-tropic HIV infections.

## 5 Conclusion

Current treatment approaches for HIV infection are generally limited to medications that target various viral enzymes, although a few do target viral entry, namely Maraviroc, Ibalizumab, Temsavir, and Enfuvirtide. No FDA- or EMA-approved medication addresses the fact that CCR2 contributes to the migration of immune cells and, in some cases, provides an alternative route to HIV infection (85), which potentially augments the clinical importance of targeting CCR2. Whether modulation of hnRNPs, including hnRNPA0, could ever impact HIV disease and progression, inflammatory processes, and malignancy, remains to be explored. Elucidating the mechanisms behind CCR2 post-transcriptional regulation may provide a basis for developing novel therapeutic strategies against these processes. Our findings highlight the importance of post-transcriptional control in fine-tuning gene expression and its important in maintaining homeostasis in the immune system.

The binding of RNA-binding proteins (RBPs) to the 3′-UTR may contribute significantly to both the temporal and spatial regulation of gene expression. This is particularly relevant for diseases where dysregulated expression contributes to pathology. The widespread expression of CCR5 and the more restricted, inflammation-inducible expression of CCR2 suggest that these receptors are regulated not only at the transcriptional level but also through post-transcriptional mechanisms, including mRNA stability. Our findings demonstrate that deletion of the CCR2 3’-UTR significantly increases mRNA half-life, indicating that this region normally acts to destabilize CCR2 transcripts. This effect is likely mediated by specific cis-regulatory elements and their interaction with inhibitory RBPs, such as hnRNPA0. Understanding these mechanisms expands our knowledge of immune gene regulation and highlights the potential for targeting 3’-UTR-RBP interactions as a therapeutic strategy in conditions such as chronic inflammation, autoimmunity, and HIV infection.

## Data Availability

The original contributions presented in the study are included in the article/**Supplementary Material**. Further inquiries can be directed to the corresponding author.

## Supplementary material

Supplementary Figure 1 Construction of CCR2 3’-UTR subclones (FrA, FrB, FrC, FrD, and FrE). **(A)** The pGL3-CMV-FFLUC plasmid backbone is depicted, including key elements such as the CMV promoter, firefly luciferase gene, and XbaI cloning site in which each DNA segment was inserted (magenta). **(B)** The full-length CCR2 3’-UTR construct is shown, highlighting its placement into the pGL3-Basic-CMV-FFLUC vector. **(C)** Subclones FrA to FrE are displayed with their respective lengths and positions within the CCR2 3’-UTR. (D) FrC derivatives (FrC1 to FrC4) are illustrated, focusing on shorter fragments within the FrC region. All subclones were either PCR-amplified from the FlUTR plasmid or cloned using restriction enzymes into the pCR-Blunt II-TOPO plasmid and pGL3-Basic-CMV- FFLUC reporter vector, respectively.

Supplementary Figure 2 Flow cytometric analysis of CCR2 and CCR5 expression in GHOST.CCR2B cells. Cells were stained with anti-CCR2-PE **(A)** or anti-CCR5-APC **(B)** antibodies and then subjected to flow cytometry, confirming presence of CCR2 and absence of CCR5 cell surface expression.

Supplementary Figure 3 Flow cytometry of CCR2 for KO KHSRP, KO RALY cells and KO HnRNPDL. After KO of KHSRP, RALY, and HnRNPDL, when compared to mock KO, changes in CCR2 cell surface expression were not significant (ns).

Supplementary Figure 4 CBB Staining of GST and GST-HnRNPA0 fusion proteins. E. coli was transformed with plasmids encoding GST (lane 1) or GST-hnRNPA0 (lane 2) and GST proteins purified using glutathione beads prior to SDS-PAGE and CBB staining/destaining. Size of GST is ~26 kDa, whereas the GST-hnRNPA0 fusion is ~57 kDa.

Supplementary Figure 5 Validation of Knockout Efficiency for CCR2 3′-UTR and RNA-Binding Proteins (RBPs). **(A)** Gene expression analysis of RBP targets (hnRNPA0, hnRNPDL, RALY, and KHSRP) following CRISPR-Cas9 knockout. Total RNA was extracted 72 hours post-electroporation, and relative mRNA levels were quantified by qPCR using gene-specific primers. Knockdown efficiency was calculated relative to mock-transfected controls. Data represent mean ± SD from three independent experiments. **(B)** Knockout efficiency of CCR2 3′-UTR was assessed by nested PCR using genomic DNA extracted from primary CD4^+^ T cells following CRISPR-Cas9 editing. PCR products were visualized on agarose gels, and densitometry analysis indicated an average knockout efficiency of ~50%. Lane 1: Control, Lane 2: KO CCR2 3`UTR.
