# Supplementary figures and images for: The role of the 3′-UTR of the chemokine receptor CCR2 and hnRNPA0 in regulating mRNA stability and subcellular distribution in human CD4+ T cells
Source: Front Immunol. 2025 Aug 20;16:1655273. doi: 10.3389/fimmu.2025.1655273 (PMC12405378; doi:10.3389/fimmu.2025.1655273)

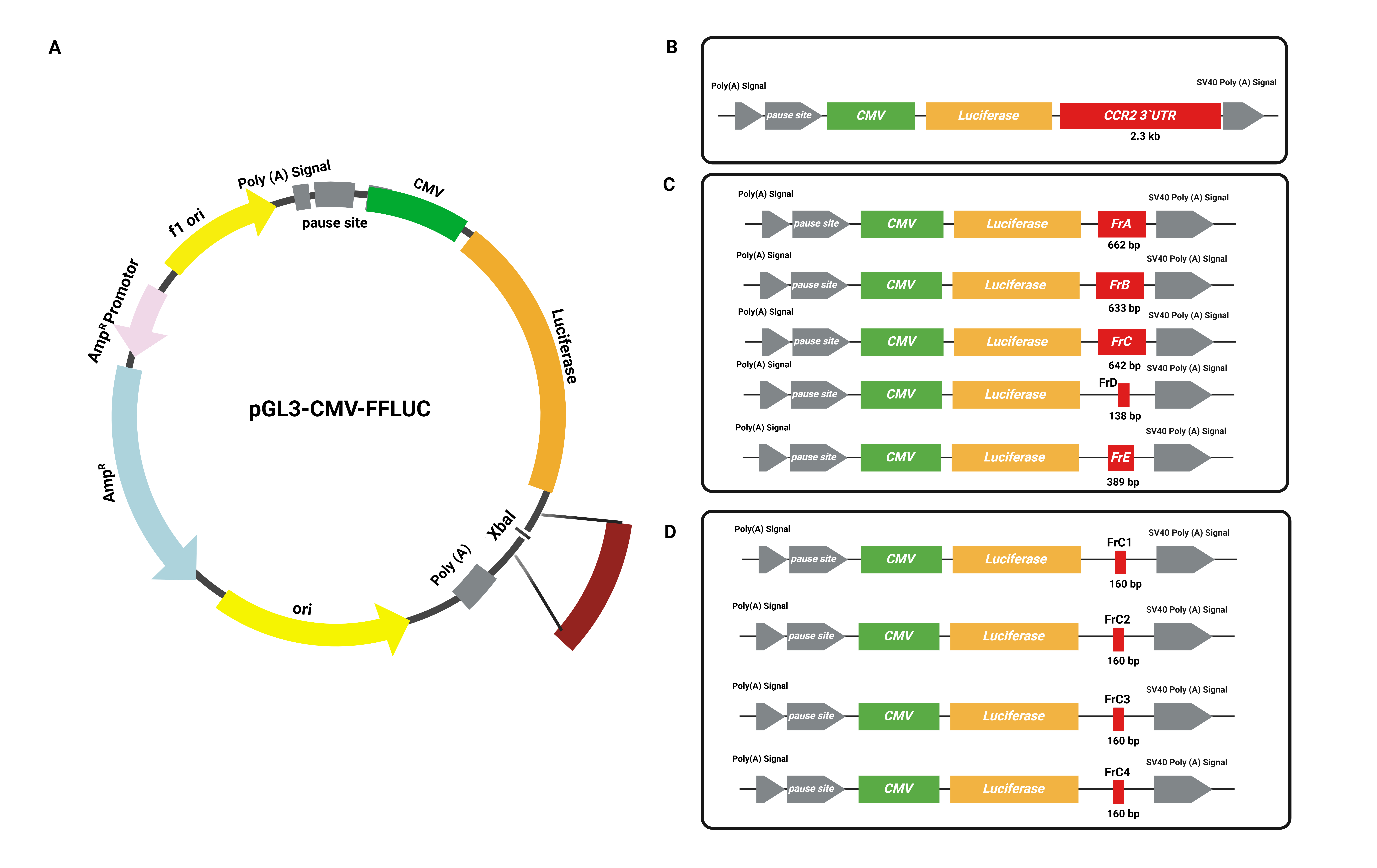

Supplement: Supplementary Figure 1 — Construction of CCR2 3’-UTR subclones (FrA, FrB, FrC, FrD, and FrE). (A) The pGL3-CMV-FFLUC plasmid backbone is depicted, including key elements such as the CMV promoter, firefly luciferase gene, and XbaI cloning site in which each DNA segment was inserted (magenta). (B) The full-length CCR2 3’-UTR construct is shown, highlighting its placement into the pGL3-Basic-CMV-FFLUC vector. (C) Subclones FrA to FrE are displayed with their respective lengths and positions within the CCR2 3’-UTR. (D) FrC derivatives (FrC1 to FrC4) are illustrated, focusing on shorter fragments within the FrC region. All subclones were either PCR-amplified from the FlUTR plasmid or cloned using restriction enzymes into the pCR-Blunt II-TOPO plasmid and pGL3-Basic-CMV- FFLUC reporter vector, respectively. [file Image1.jpeg]

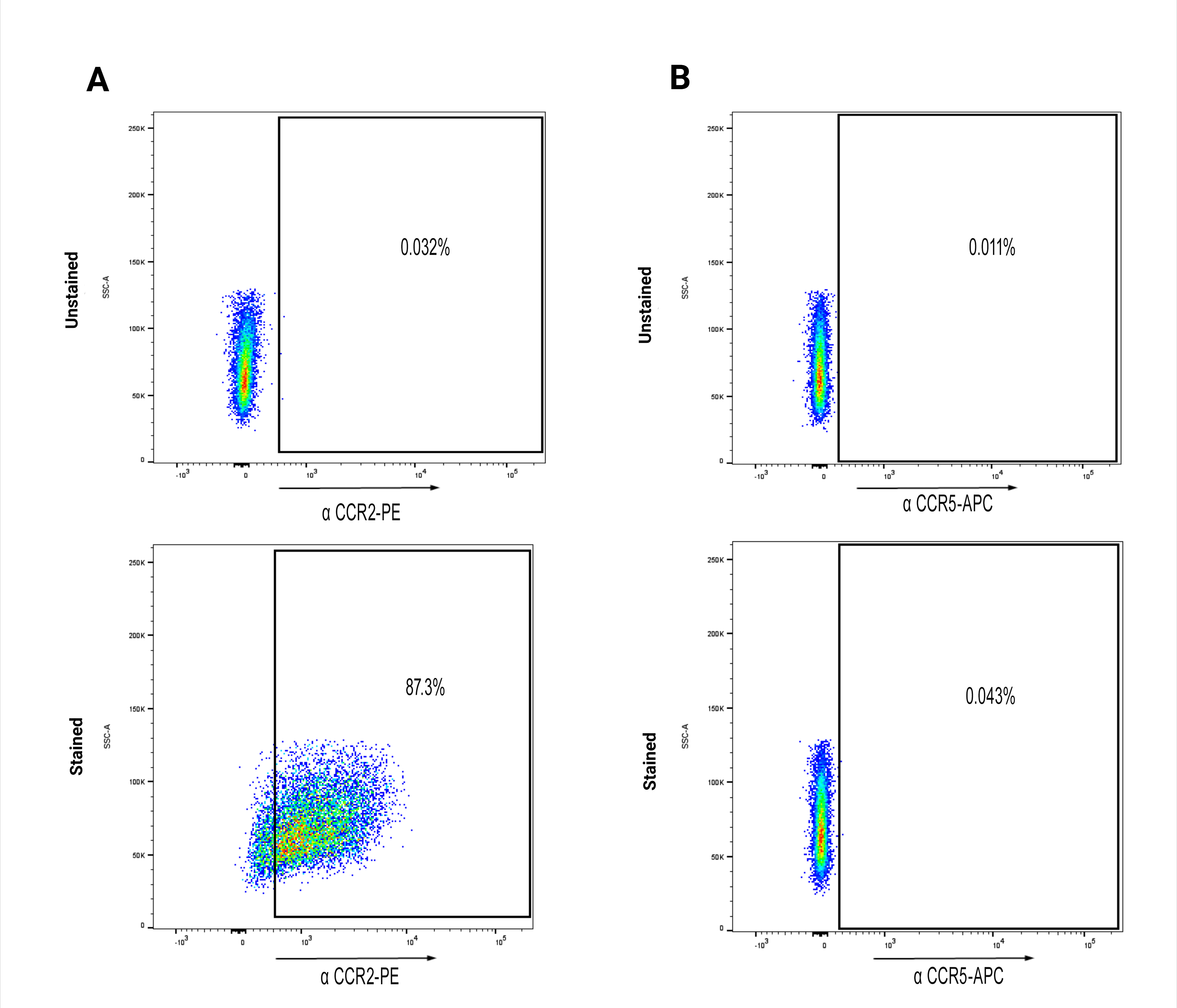

Supplement: Supplementary Figure 2 — Flow cytometric analysis of CCR2 and CCR5 expression in GHOST.CCR2B cells. Cells were stained with anti-CCR2-PE (A) or anti-CCR5-APC (B) antibodies and then subjected to flow cytometry, confirming presence of CCR2 and absence of CCR5 cell surface expression. [file Image2.jpeg]

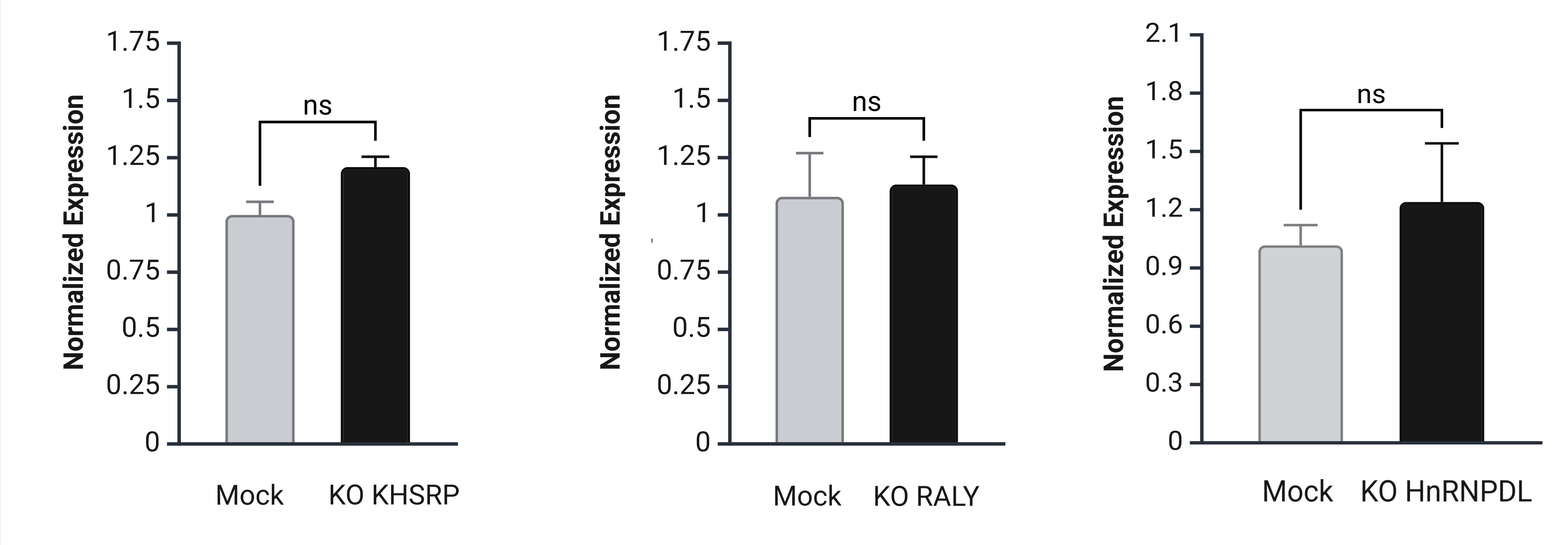

Supplement: Supplementary Figure 3 — Flow cytometry of CCR2 for KO KHSRP, KO RALY cells and KO HnRNPDL. After KO of KHSRP, RALY, and HnRNPDL, when compared to mock KO, changes in CCR2 cell surface expression were not significant (ns). [file Image3.jpeg]

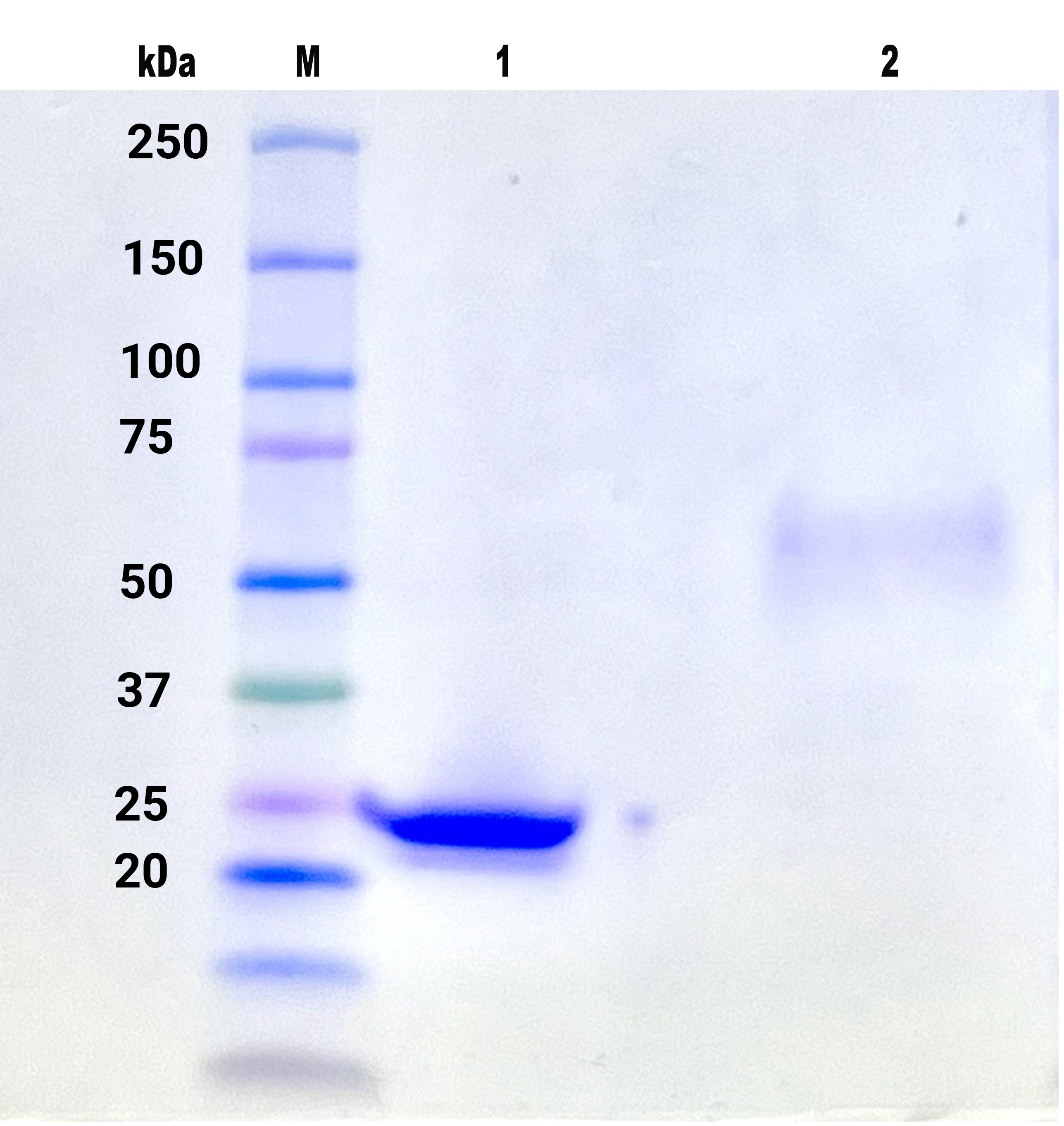

Supplement: Supplementary Figure 4 — CBB Staining of GST and GST-HnRNPA0 fusion proteins. E. coli was transformed with plasmids encoding GST (lane 1) or GST-hnRNPA0 (lane 2) and GST proteins purified using glutathione beads prior to SDS-PAGE and CBB staining/destaining. Size of GST is ~26 kDa, whereas the GST-hnRNPA0 fusion is ~57 kDa. [file Image4.jpeg]

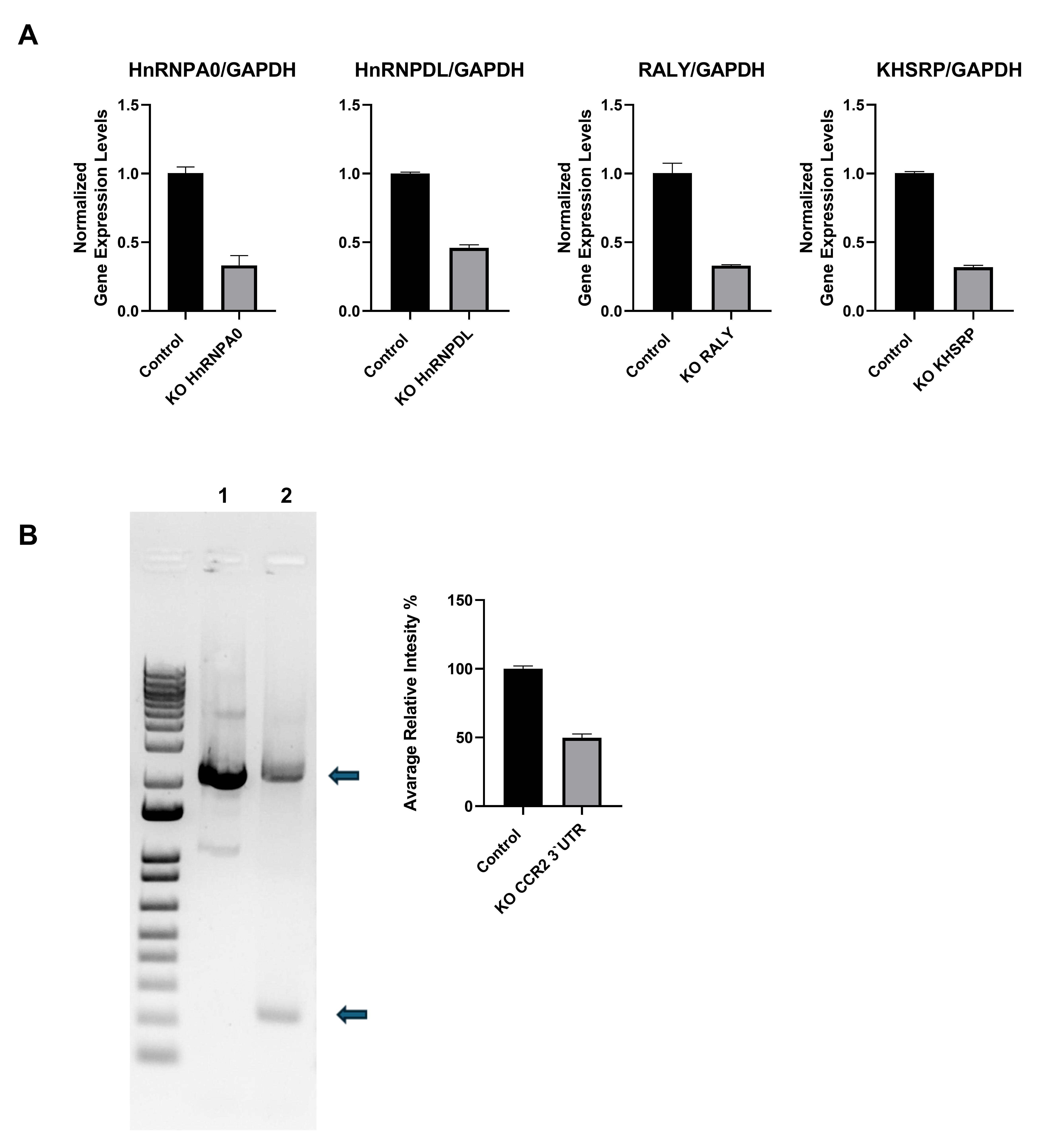

Supplement: Supplementary Figure 5 — Validation of Knockout Efficiency for CCR2 3′-UTR and RNA-Binding Proteins (RBPs). (A) Gene expression analysis of RBP targets (hnRNPA0, hnRNPDL, RALY, and KHSRP) following CRISPR-Cas9 knockout. Total RNA was extracted 72 hours post-electroporation, and relative mRNA levels were quantified by qPCR using gene-specific primers. Knockdown efficiency was calculated relative to mock-transfected controls. Data represent mean ± SD from three independent experiments. (B) Knockout efficiency of CCR2 3′-UTR was assessed by nested PCR using genomic DNA extracted from primary CD4+ T cells following CRISPR-Cas9 editing. PCR products were visualized on agarose gels, and densitometry analysis indicated an average knockout efficiency of ~50%. Lane 1: Control, Lane 2: KO CCR2 3`UTR. [file Image5.jpeg]
