# Supplementary material for: The role of the 3′-UTR of the chemokine receptor CCR2 and hnRNPA0 in regulating mRNA stability and subcellular distribution in human CD4+ T cells
Source: Front Immunol. 2025 Aug 20;16:1655273. doi: 10.3389/fimmu.2025.1655273 (PMC12405378; doi:10.3389/fimmu.2025.1655273)
Supplement: Supplementary file 6 [file Table1.docx]

**Table S1 ; List of Reagents, Primers, and Experimental Tools with Corresponding Sources and Catalog Numbers**

| **Reagent / Resource** | **Reference or Source** | **Identifier or catalog number** |
| --- | --- | --- |
| **Antibodies** | | |
| Anti-human CD4-FITC (clone SK3) | BioLegend | Cat#344604 |
| Anti-CCR2-PE (clone K036C2) | BioLegend | Cat# 357206 |
| Purified Anti-CD3 | BioLegend | Cat# 830301 |
| Purified Anti-CD28 | BioLegend | Cat# 302902 |
| Anti-CCR5-APC (clone J418F1) | BioLegend | Cat# 359122 |
| **Bacterial and virus strains** | | |
| VSV-G pseudotyped HIV | Sutton lab |  |
| HIV-cycT1-IRES-YFP | Sutton lab |  |
| pSRα-YU2 | Sutton lab |  |
| Env ADA | Sutton lab |  |
| Biological samples | | |
| Human PBMCs | New York Blood Center |  |
| Primary CD4+ T cells | Isolated from PBMCs |  |
| Primary macrophages | Differentiated from PBMCs |  |
| **Chemicals, peptides, and recombinant proteins** | | |
| Recombinant human IL-2 | STEMCELL Technologies | Cat#78036.3 |
| Recombinant human IL-4 | Sino Biological | Cat#GMP-11846-HNAE |
| Recombinant human IL-10 | Sino Biological | Cat#10947-HNAE |
| Recombinant M-CSF | BioLegend | Cat#574802 |
| GST-hnRNPA0 fusion protein | Produced in-house | N/A |
| GST protein | Produced in-house | N/A |
| DMEM | GIBCO | Cat#11965118 |
| Heat inactivated Fetal Bovine Serum | Gemini Bio | Cat#100-106G-500 |
| Amphotericin B | Gemini Bio | Cat#400104/100 |
| Dimethyl sulfoxide | Sigma | Cat#D2650 |
| Penicillin-streptomycin solution | GIBCO | Cat#15140148 |
| RPMI-1640 | GIBCO | Cat#11875093 |
| Trypsin-EDTA | GIBCO | Cat#25200056 |
| Luria Broth (LB) | This Paper | N/A |
| Biotin-16-UTP | Roche | Cat# 11388908910 |
| **Critical commercial assays** | | |
| EasySep Human CD4+ T Cell Isolation Kit | STEMCELL Technologies | Cat# 100-0696 |
| Dual-Luciferase Reporter Assay Kit | Promega | Cat# E1960 |
| SuperScript™ III Platinum™ SYBR™ Green One-Step qRT-PCR Kit | ThermoFisher Scientific | Cat # 11736059 |
| Zero Blunt™ TOPO™ PCR Cloning Kit, | ThermoFisher Scientific | Cat# 451245 |
| Neon™ Transfection System 10 μL Kit | ThermoFisher Scientific | Cat#MPK1025 |
| VIVID, LIVE/DEAD Viability/Cytotoxicity Kit | ThermoFisher Scientific | Cat#L34955 |
| NE-PER™ Nuclear and Cytoplasmic Extraction Reagents | ThermoFisher Scientific | Cat# 78833 |
| **Experimental models: Cell lines** | | |
| Human GHOST (3) CCR2b+ Cells | BEI Resource NIH AIDS Research and Reference Program | Cat# ARP-3681 |
| Human GHOSTHi CCR5 Cells | BEI Resource NIH AIDS Research and Reference Program | Cat# ARP-3944 |
| 293T | ATCC | Cat# CRL-3216 |
| **Experimental models: Organisms/strains** | | |
| **Oligonucleotides** | | |
| **qPCR CCR2 Primers;**  Forward GACTGACATTTACCTGCTCAACC  Reverse AAATAACCGATGTGATACAGCCCT | This paper |  |
| **qPCR GAPDH Primers;**  Forward TTTTGCGTCGCCAGCC  Reverse ATGGAATTTGCCATGGGTGGA | This paper |  |
| **qPCR hnRNPA0 primers;**  Forward GGTTAAGAAGCTCTTTGTCGG  Reverse TGAATCGGATGGAACTTGAC | This paper |  |
| **qPCR hnRNPDL primers;**  Forward CTGCCGACAGCTCCGTCA  Reverse ACAACTTCCCCAAATCGAGAC |  |  |
| **Site Directed Mutagenesis Del1 in FrC**  **Forward**  CAAACTACCTTCCAGTTCCTC AATACAGGCA  **Reverse** CTCTATGCCTGTATTGAGGAACTGGAAGGTAG | This Paper |  |
| **Site Directed Mutagenesis Del2 in FrC**  **Forward**  TGAATACAGGCATAGAGTTCAG ATAGTAAAAATA  **Reverse**  AATTTTATTTTTACTAT CTGAACTCTATGCCTGTATT | This Paper |  |
| **Site Directed Mutagenesis Mid Del in FrC**  **Forward** CAGTTCCTCATTTTTGATTCAGACTTTTTTTAA  **Reverse**  TTACTATTTAAAAAAAGTCTGAATCAAAAATGAGGA | This Paper |  |
| **qPCR MALAT-1 primers**  Forward CTGACCCAGGTGCTACACAG  Reverse GCTTGCTCCTCAGTCCTAGC | This Paper |  |
| **qPCR RPL30 primers**  Forward CACCAGTTTTAGCCAACATAGC  Reverse TGAAGATGATCAGACAAGGCAA | This Paper |  |
| **Upstream Cloning of FrC Primers**  **Forward** : smaI cccggg CACAGATGTGTGATTCAC  **Reverse:** XhoI ctcgag AAAAGCTTTGATTAGAAGCCAACTTGATTTAGG | This Paper |  |
| **Fragment C Subclones Primers**  **Fragment C1**  Forward XbaI TCTAGA CACAGATGTGTGATTCACAG  Reverse XbaI TCTAGA TTTAAAAAAAGTCTGAACTCTATGC  **Fragment C2**  Forward XbaI TCTAGA TAGTAAAAATAAAATTAAAGCTGAAAACTG  Reverse XbaI TCTAGA AGTCTCCCAAACATACCAC  **Fragment C3**  Forward XbaI TCTAGA GCTGAGTCAACCCAATAG  Reverse XbaI TCTAGA AGCCTGAGAGAATGGAG  **Fragment C4**  Forward XbaI TCTAGA TGCTGCCAAAAGCCTT  Reverse XbaI TCTAGA AAAAGCTTTGATTAGAAGCCAA | This Paper |  |
| **Full Length 3`UTR Subclones Primers**  **Fragment A**  Forward AACGAGGAGCAGTTTGATTGT  Reverse ATGGGTGCCATAGATAAACTGT  **Fragment B**  Forward GCACCTTACATTTGAAATCTATGAAAT  Reverse AAGCCAGACGTGTGATTTCC  **Fragment C**  Forward CACAGATGTGTGATTCACAGT  Reverse AAAAGCTTTGATTAGAAGCCAAC  **Fragment D**  Forward AAACCCTATTGGTAAAGAATGGAAG  Reverse AGCCCAGTGTTTCTTGCTTGCATC  **Fragment E**  Forward ACCAGGCAACTTGGGAACTAGACTC  Reverse TTGTTTTTAAATCTTTTATCAACATAGTTATAGC | This Paper |  |
| **CCR2 3’-UTR was PCR amplified from an existing BAC clone and cloned using pCR primers**  Forward AACGAGGAGCAGTTTGATTG  Reverse AGTTGTAAAAAAACAGCCAGTTG | This Paper |  |
| **GST cloning hnRNPA0 PCR primers**  **Forward**  SalI GTCGACAATGGAGAATTCTCAGTTGTGTAAG  **Reverse**  NotIGCGGCCGCGAAGGAGCTGCCTCCATA | This Paper |  |
| **CCR2 3`UTR KO DNA Nested PCR Primers**  **Out Forward ;**  AACGAGGAGCAGTTTGATTGTT  **Out Reverse;**  ACAGTATTTACATTTCATCACCATCAACTTA  **In Forward;**  TAACAACAAACTTCAAGGGTTTGTTG  **In Reverse;**  TCATTAGGATCATACAAATATATGAAGGTGAAAGT | This paper |  |
| **CCR2 3`UTR Knockout**  sgRNA 1 CTTGATTTAGGAGTTCCCAC  sgRNA 2 ATCTTGGTGTCTACGTTACC | IDT |  |
| **hnRNPA0 Knockout**  sgRNA1: CGTGACCTACTCCAATGTGG | IDT |  |
| **hnRNPDL Knockout**  sgRNA1: GAACGAGTACAGCAATATAG | IDT |  |
| **KHSRP Knockout**  sgRNA1: AGTGCTGTTATTCACTGTCG | IDT |  |
| **Recombinant DNA** | | |
| pGEX-5X-2 | Addgene | Cat#27-4584-01 |
| pGL3 Basic Vector | Addgene | Cat#212936 |
| pMaxGFP | Addgene | Cat#177825 |
| CCR2 3'-UTR FlUTR reporter constructs | This paper |  |
| CCR2 3'-UTR FrA reporter constructs | This paper |  |
| CCR2 3'-UTR FrB reporter constructs | This paper |  |
| CCR2 3'-UTR FrC reporter constructs | This paper |  |
| CCR2 3'-UTR FrD reporter constructs | This paper |  |
| CCR2 3'-UTR FrE reporter constructs | This paper |  |
| CCR2 3'-UTR FrC1 reporter constructs | This paper |  |
| CCR2 3'-UTR FrC2 reporter constructs | This paper |  |
| CCR2 3'-UTR FrC3 reporter constructs | This paper |  |
| CCR2 3'-UTR FrC4 reporter constructs | This paper |  |
| Site Directed Mutagenesis FrC 7bp Del1 constructs | This paper |  |
| Site Directed Mutagenesis FrC 14bp MidDel constructs | This paper |  |
| Site Directed Mutagenesis FrC12bp Del2 constructs | This paper |  |
| Upstream CCR2 3`UTR FrC reporter constructs | This paper |  |
| GST-fusion hnRNPA0 construct | This Paper |  |
| Software and algorithms | | |
| ImageJ | Schneider et al.2012 | https://imagej.nih.gov/ij/ |
| GraphPad Prism | GraphPad Software | Version 9.0 |
| FlowJo | BD Biosciences | Version 10.9 |
